# Transcriptomic entropy benchmarks stem cell-derived cardiomyocyte maturation against endogenous tissue at single cell level

**DOI:** 10.1371/journal.pcbi.1009305

**Published:** 2021-09-17

**Authors:** Suraj Kannan, Michael Farid, Brian L. Lin, Matthew Miyamoto, Chulan Kwon

**Affiliations:** 1 Division of Cardiology, Department of Medicine, Johns Hopkins School of Medicine; Baltimore, Maryland, United States of America; 2 Department of Biomedical Engineering, Johns Hopkins School of Medicine; Baltimore, Maryland, United States of America; 3 Institute for Cell Engineering, Johns Hopkins School of Medicine; Baltimore, Maryland, United States of America; University of Virginia, UNITED STATES

## Abstract

The immaturity of pluripotent stem cell (PSC)-derived tissues has emerged as a universal problem for their biomedical applications. While efforts have been made to generate adult-like cells from PSCs, direct benchmarking of PSC-derived tissues against *in vivo* development has not been established. Thus, maturation status is often assessed on an *ad-hoc* basis. Single cell RNA-sequencing (scRNA-seq) offers a promising solution, though cross-study comparison is limited by dataset-specific batch effects. Here, we developed a novel approach to quantify PSC-derived cardiomyocyte (CM) maturation through transcriptomic entropy. Transcriptomic entropy is robust across datasets regardless of differences in isolation protocols, library preparation, and other potential batch effects. With this new model, we analyzed over 45 scRNA-seq datasets and over 52,000 CMs, and established a cross-study, cross-species CM maturation reference. This reference enabled us to directly compare PSC-CMs with the *in vivo* developmental trajectory and thereby to quantify PSC-CM maturation status. We further found that our entropy-based approach can be used for other cell types, including pancreatic beta cells and hepatocytes. Our study presents a biologically relevant and interpretable metric for quantifying PSC-derived tissue maturation, and is extensible to numerous tissue engineering contexts.

## Author summary

There is significant interest in generating mature cardiomyocytes from pluripotent stem cells. However, there are currently few effective metrics to quantify the maturation status of a single cardiomyocyte. We developed a new metric for measuring cardiomyocyte maturation using single cell RNA-sequencing data. This metric, called entropy score, uses the gene distribution to estimate maturation at the single cell level. Entropy score enables comparing pluripotent stem cell-derived cardiomyocytes directly against endogenously-isolated cardiomyocytes. Thus, entropy score can better assist in development of approaches to improve the maturation of pluripotent stem cell-derived cardiomyocytes.

This is a *PLOS Computational Biology* Methods paper.

## Introduction

The development of robust protocols for differentiation of pluripotent stem cells (PSCs) to a range of somatic tissues has represented a huge advance in biomedical research over the past two decades. Many somatic cell types are non-proliferative and difficult to obtain from patients, and thus PSCs may be the most viable source for generating large quantities of specialized tissue. PSC-derived tissues have numerous promised applications in regenerative medicine, drug efficacy and toxicity screening, and *in vitro* disease modeling [1–5]. However, clinical application of PSC-derived tissues has been limited thus far due to the failure of these cells to mature to a fully adult-like phenotype *ex vivo*. This phenomenon has been observed in a range of engineered tissue types, including cardiomyocytes (CMs) [6], hepatocytes [7], pancreatic islet cells [8], neurons [9], and others, and represents a major biomedical hurdle.

To date, numerous engineering approaches have been proposed to improve PSC-derived tissue maturation. These approaches have included cocktails, induction of physical stimuli, co-culture with other cells, and construction of three-dimensional tissues, typically with the goal of recapitulating the native milieu [6,8,10–15]. Benchmarking the efficacy of these interventions has been challenging, however, as functional assays for direct comparison of PSC-derived cells to endogenous adult cells are often technically infeasible. Thus, most engineered tissues are compared either to a two-dimensional *in vitro* control or at best one discrete (usually neonatal) *in vivo* timepoint, rather than across the continuous spectrum of *in vivo* maturation. Several groups have proposed use of -omics data to compare engineered to *in vivo* tissues for certain cells [16–19]. However, these approaches have been limited to bulk samples, which precludes their use when PSC differentiation yields highly heterogeneous populations.

scRNA-seq has emerged as a powerful tool for measuring the transcriptomes of large numbers of single cells, and is an intriguing candidate for new metrics of tissue maturation. Unfortunately, differences in isolation protocols, library preparation methods, and sequencing machines, among other factors, can imbue scRNA-seq data with batch effects that are difficult to deconvolve [20,21]. In turn, this makes it difficult to directly compare expression of individual genes across datasets. While batch correction algorithms have been developed [22], they are primarily designed for correcting or integrating datasets with multiple well-defined cell types with significantly different gene expression patterns rather than one continuously evolving cell type. Thus, an optimal scRNA-seq-based metric of maturation must facilitate direct comparison of maturation status while being robust to batch effects.

Here, we developed an approach based on quantifying gene *distributions* to assess PSC-derived tissue maturation. Given the significant burden of cardiac disease [23], we focused our analysis on CMs, the primary contractile cells of the heart. Our approach is based on the generally-observed phenomenon that less differentiated cells are typically more promiscuous in their transcriptional activities of signaling pathways, leading to a diverse gene expression profile. However, as they differentiate, they prune unnecessary signaling pathways and hone in on a relatively narrow gene expression profile [24]. This observation has been leveraged in several previous approaches to study differentiation of stem cells to progenitors and subsequently to committed lineages [25–30]. We applied this principle to study the maturation of committed CMs by developing a metric based on a modification of the Shannon entropy of scRNA-seq gene expression data. Our transcriptomic entropy-based metric not only adequately stages single CMs, but that scores are consistent across datasets regardless of potentially confounding batch effects. Using datasets from the literature, we performed a meta-analysis of CM maturation based on transcriptomic entropy. We subsequently demonstrated the use of our approach to infer the maturation status of PSC-CMs. While our primary focus was on CMs, we also showed initial evidence of applicability to other celltypes, in particular pancreatic beta cells and hepatocytes. These results establish transcriptomic entropy as a viable metric for benchmarking PSC-derived tissue maturation.

## Results

### scRNA-seq CM reference captures maturation-related changes

**Fig 1:**
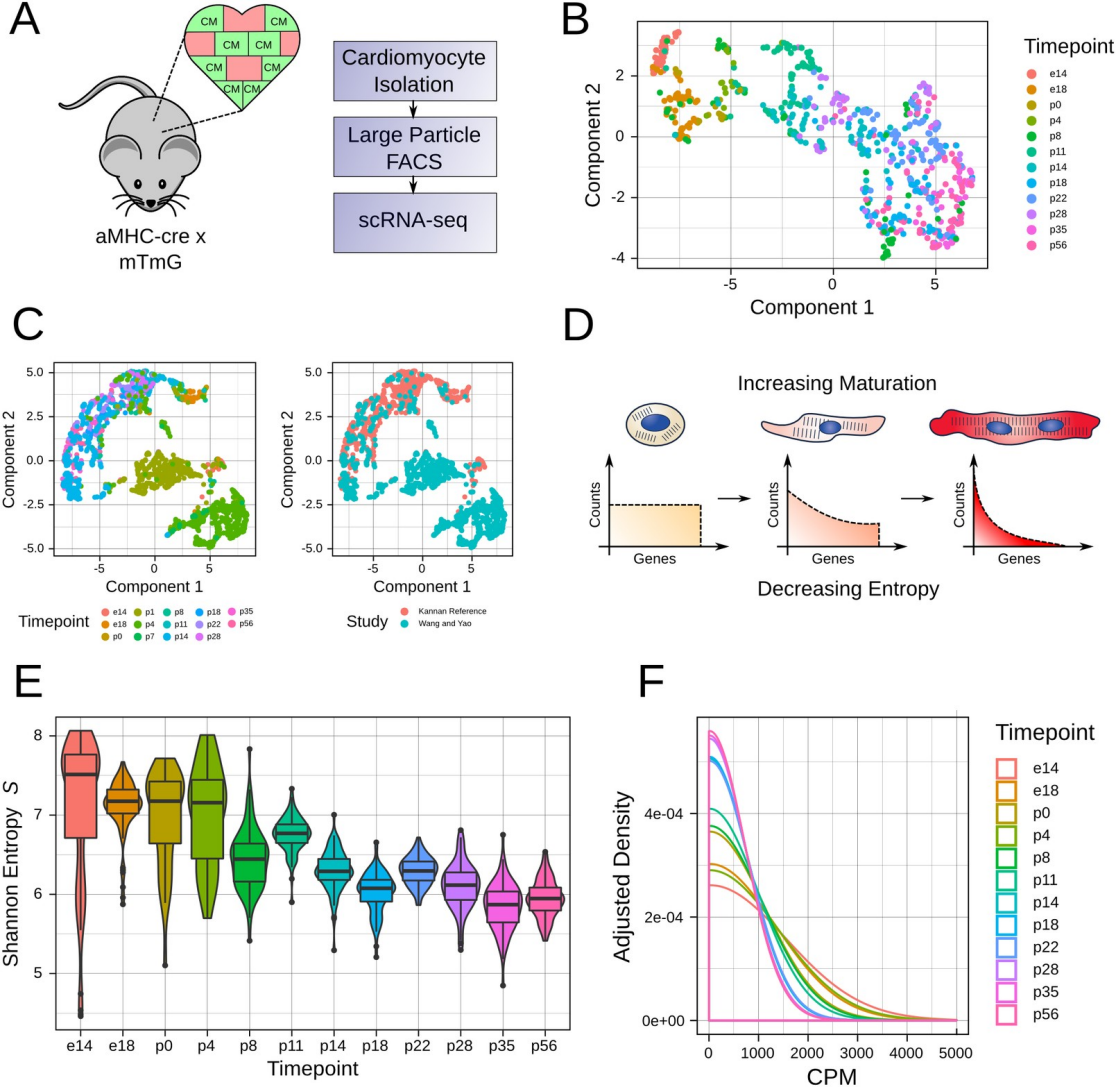
scRNA-seq constructs a reference for CM maturation. **A.** Mouse model used to generate perinatal maturation reference scRNA-seq library. In the aMHC-cre x mTmG mouse, CMs are labeled by GFP. This image was obtained and modified from “Brown Mouse Lab” by SVG-Clipart.com under a CC BY 4.0 license. **B.** UMAP dimensionality reduction (via Monocle 3) for the maturation reference. **C.** mnnCorrect-based integration of Wang and Yao et al. dataset with reference dataset. **D**. Our model for changes in gene distribution over CM maturation. As CMs undergo the maturation process, they transition from a broad gene distribution (characterised by high entropy) to a more narrow distribution (characterised by low entropy). **E**. Shannon Entropy S computed for each timepoint in the maturation reference dataset. **F.** Smoothed density estimates for genes expressed at 0–5000 counts per million (CPM) for each timepoint in the maturation reference dataset.

As a first step, we sought to construct a reference scRNA-seq library for CM maturation. Sequencing of postnatal CMs, which are large and fragile, has been previously limited [31]. Recently, however, we developed a method to isolate healthy adult CMs to generate high quality scRNA-seq libraries using large-particle fluorescence-activated cell sorting (LP-FACS) [32]. We used this approach to isolate CMs from *Myh6-Cre; mTmG* (aMHC x mTmG) mice, in which cells expressing cardiac-specific myosin heavy chain are readily separated by GFP expression **(Fig 1A)**. We generated a library of ~1000 CMs from 12 points over the course of maturation. Our reference particularly sampled cells within the first three weeks postnatally, as this period may be critically relevant to the maturation process but is underrepresented in existing CM scRNA-seq datasets.

Dimensionality reduction of our reference with uniform manifold approximation and projection (UMAP) revealed a continuum of maturation from e14 in p56, in concordance with previous results from our group and others [33–35] **(Fig 1B)**. As an initial strategy, we considered quantification of maturation status by integration of query datasets with our reference. We tested this approach by using mnnCorrect [36] to combine other *in vivo* CM scRNA-seq datasets with our reference while correcting study-specific batch effects. While this approach worked for some datasets, in other cases study-dependent batch-effects were only partially corrected **(Fig 1C)**. Moreover, integration often changed cell-to-cell distances within our original reference itself. These results confirmed the difficulty of quantifying CM maturation through a conventional dimensionality reduction/batch correction-based pipeline, and prompted us to seek other approaches for quantifying maturation from scRNA-seq data.

### Shannon entropy of single cell gene expression decreases over CM maturation

We next considered an approach based on gene distribution changes. Following terminal differentiation, CMs undergo a lengthy maturation process characterized by gradual and unidirectional changes in gene expression [17]. Based on previous findings, we proposed a model for transcriptional maturation of CMs analogous to cellular differentiation **(Fig 1D)**. In this model, nascent cardiomyocytes express a broad gene expression profile. However, as they mature, they slowly reduce expression of immature gene pathways (e.g. cell cycle) while upregulating genes required for mature function (e.g. sarcomere, calcium handling, oxidative phosphorylation). These gradual changes in gene distribution can be quantified by established diversity metrics such as the well-known Shannon entropy. In our model, immature myocytes will present with high transcriptomic entropy, which subsequently decreases in a continuous manner over the course of maturation.

To test the validity of this model, we computed the Shannon entropy *S* on the unique molecular identifier (UMI) counts of our maturation reference **(Fig 1E)**. Entropy gradually decreased from e14 to p56, with a notable shift from p4 to p8, thereby supporting our hypothesized entropy model. We additionally plotted the averaged gene distributions for each timepoint **(Fig 1F)**. As expected, earlier timepoints showed a more broad distribution compared to later timepoints. These results supported the use of Shannon entropy to quantify CM maturation status from scRNA-seq data.

### Gene and cell filtration are necessary for cross-study comparison

Given the correspondence between Shannon entropy and CM maturation status, we next sought to determine whether we could extend our transcriptomic entropy model to many CM scRNA-seq datasets generated across multiple labs. We identified publicly available scRNA-seq datasets containing CMs isolated *in vivo* **(S1 Table)**. Our meta-analysis included 34 mouse datasets and 5 human datasets spanning numerous timepoints across the range of development. Additionally, the collected datasets represented significant diversity in terms of isolation methods, sequencing protocols, mapping/counting pipelines, and datatypes (including reads from full-length scRNAseq protocols, 3’ counts from UMI protocols prior to UMI collapsing, and UMIs). However, several technical challenges prevented accurate cross-study comparison of Shannon entropy computed on raw, unfiltered datasets. These particular challenges and our solutions are addressed here.

#### Handling multiple mapping/counting pipelines

One problem that was observed in certain sequencing mapping/counting pipelines was the incorrect mismapping of mitochondrial reads to pseudogenes. In mice, fragments of the mitochondrial genome are present as pseudogenes in the nuclear genome (termed nuclear mitochondrial insertion sequences [37]). These fragments often show identical or near-identical sequences to mitochondrial genes. Thus reads are often multi-mapped between canonical mitochondrial genes and pseudogenes, leading to inaccurate gene quantification in pipelines counting multi-mapping reads. This issue was particularly problematic for CMs, as they naturally express high amounts of mitochondrial genes [32].

**Fig 2:**
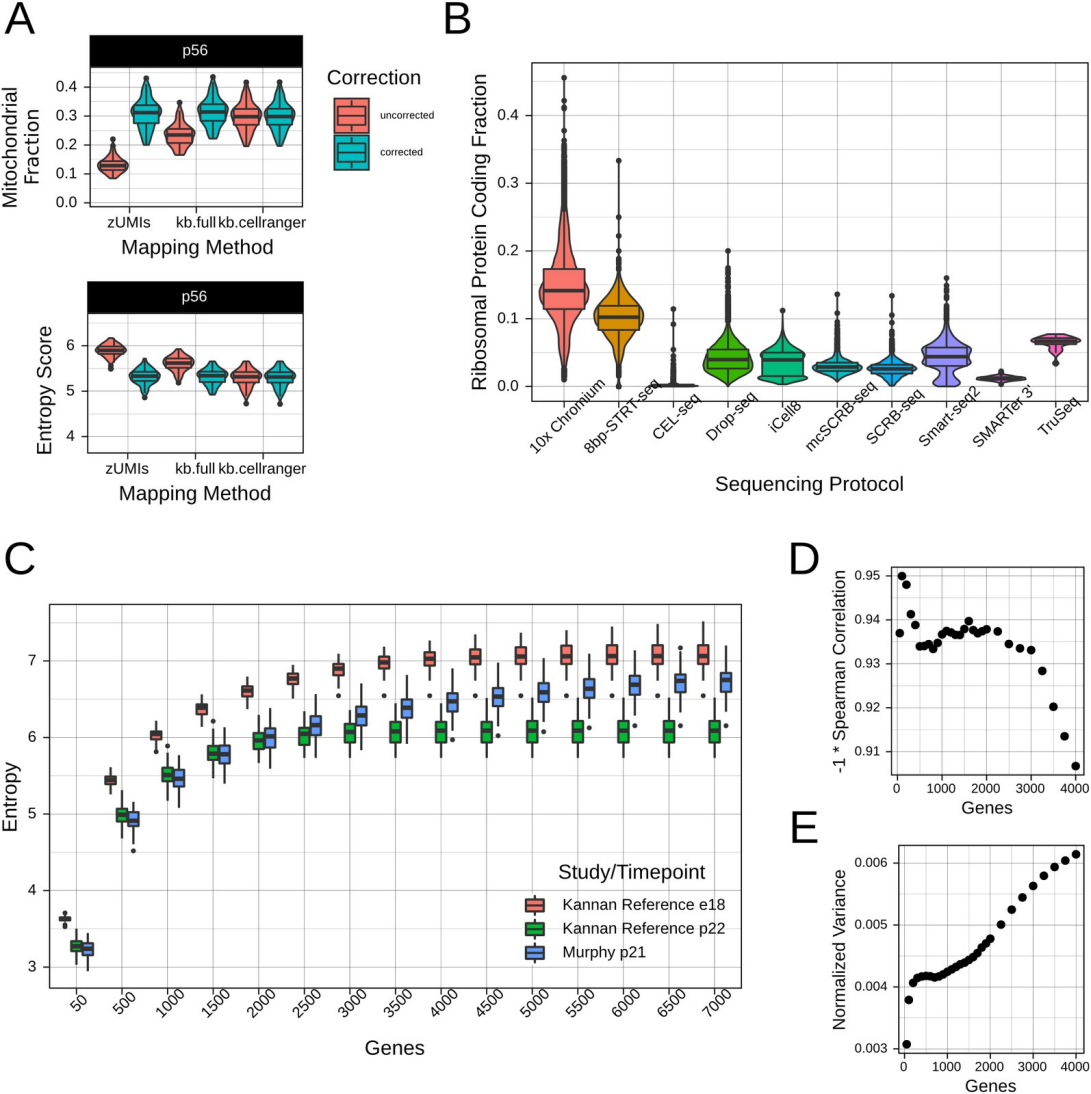
Gene filtration facilitates cross-study comparisons of entropy. **A**. Correction of mismapped mitochondrial reads. The use of a genomic mapping algorithm (such as zUMIs) or a “full” reference containing pseudogenes can lead to erroneous mapping of mitochondrial genes to pseudogenes, in turn inflating entropy score. We included a correction step that facilitates usage of data from a range of mapping pipelines. **B.** Proportion of ribosomal protein coding genes in mouse in vivo datasets, grouped by library preparation method. Given the clear protocol-dependence of these genes, we eliminated them from analysis. **C.** Entropy at different gene subsamplings for two studies with different sensitivities. Data from e18 and p22 from the maturation reference and p21 from Murphy et al. are shown. **D.** Spearman correlation between entropy and timepoint for different gene subsamplings (using median of entropy for each timepoint and study). **E.** Normalized variance of entropy for different gene subsamplings (using median of entropy for each timepoint). We normalized by scaling entropy at every subsampling to [0, 1].

As our goal was to enable entropy to be widely usable across many protocols, we included an approximate pseudogene correction in our pipeline. We identified cross-mappings between pseudogenes and canonical genes, and subsequently removed all pseudogene counts and added them to the corresponding canonical mitochondrial genes. To test the efficacy of our pseudogene correction, we tested the entropy score and well as mitochondrial gene percentages before and after correction for several mapping/counting methods **(Figs 2A and S1)**. As a genomic method, we used the zUMIs pipeline [38], which uses STAR for mapping followed by FeatureCounts for counting. In this method, multimapping counts are effectively randomly allocated between mitochondrial reads and pseudogenes. As a transcriptomic method, we utilized kallisto|bustools [39]. We used kallisto|bustools with two indices–a full index containing all mouse cDNAs from ENSEMBL (kb.full), and an index containing only protein coding, lincRNAs, and antisense RNAs analogous to the Cell Ranger index (kb.cellranger). Lastly, we also used Cell Ranger, a part of the 10x Genomics pipeline. The Cell Ranger index does not contain pseudogenes, and thus does not feature mitochondrial read mismapping.

The results of correction are shown for p56 samples in our reference **(Fig 2A)**. Prior to correction, zUMIs and kb.full produced datasets with lower mitochondrial read percentage and therefore higher entropy. However, post-correction, these datasets showed entropy and mitochondrial read percentages that were nearly identical to kb.cellranger and Cell Ranger. Thus, datasets prepared using methods that include multi-mapping reads will be sufficiently corrected for cross-study comparison.

#### Ribosomal protein-coding genes

By default, we included for analysis genes with gene biotype “protein coding,” “antisense,” or “lncRNAs,” so as to focus on the key players of the transcriptome. We additionally considered ribosomal protein-coding genes, and found significant protocol-related biases in terms of expression of these genes **(Figs 2B and S2)**. In particular, 10x Chromium and STRT-seq datasets appeared to have systematically higher percentages of ribosomal protein-coding genes than other protocols. This observation anecdotally matches observations made by others and likely indicates a protocol bias, though we are unsure about the reason this occurs. Therefore, we removed all ribosomal protein-coding genes prior to computation of entropy.

#### Variations in study sensitivity

Different scRNA-seq datasets will invariably detect different numbers of genes as a consequence of differences in sequencing depth and sequencing protocol sensitivity [40]. However, higher sensitivity can lead to artificially higher entropy simply by inclusion of more terms in the summation. An example of this effect is shown in **Fig 2C**, where we compared our reference (median 3000 genes/cell) against our previously generated dataset (Murphy et al., median 7044 genes/cell). We thus explored subsampling of genes as a way to standardize for the summation term in entropy and therefore sensitivity differences. Selecting the optimal number of genes for subsampling required balancing two priorities. Too many genes would result in overemphasis on sensitivity differences and incorrect separation of cells with similar developmental stage, as above. However, as entropy does not scale linearly with subsampled genes, too few genes would result in compression of the dynamic range of the metric and similar scores for cells at different developmental stages. We optimized the former by seeking to maximize Spearman correlation between entropy and timepoint **(Fig 2D)**, and the latter by seeking to maximize the variance of normalized entropy across timepoints at each subsampling **(Fig 2E)**. We selected 1000 genes as a reasonable subsampling based on both sets of results, though we would have obtained comparable results for 400–1500 genes.

#### Identifying poor quality datasets

**Fig 3:**
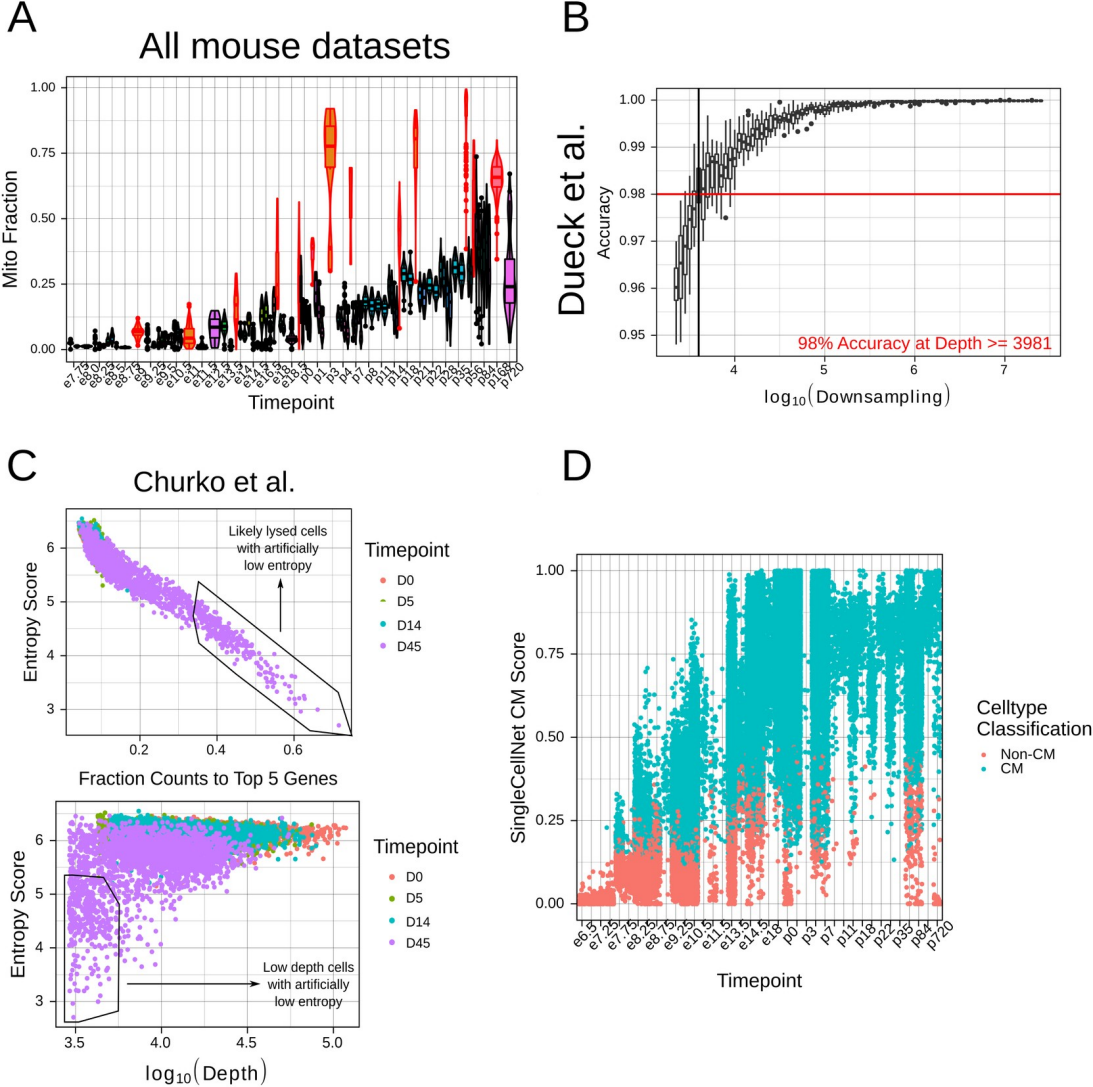
Standardized cell and study filtration enables meta-analysis of CM maturation with entropy. **A.** Mitochondrial gene fractions in mouse in vivo datasets. Datasets with unusually high proportions are highlighted in red and were removed from subsequent analysis. **B.** Subsampling of count depth in Dueck et al. dataset (the highest depth dataset in our analysis). We subsampled to a depth where the median number of genes remained > 1000. We subsequently computed the accuracy as deviation from baseline entropy. **C.** Unusually low entropy cells due to high top 5 gene percentage (top) or low depth (bottom) in the Churko et al. dataset. **D.** SingleCellNet CM scores for mouse in vivo datasets by timepoint. Cells are labeled based on whether their highest classification was for “cardiac muscle”.

Not all of the datasets identified were of sufficient quality for downstream analysis. This issue is particularly severe for CMs, where adult CMs are highly difficult to isolate at the single cell level by a number of classical methods, such as conventional FACS, single cell picking, or microfluidic devices such as the Fluidigm C1 [32]. To identify such datasets, we used the percentage of mitochondrial reads as a quality control metric, discarding datasets with unusually high percentages **(Fig 3A).** Currently, there is no automated approach for easily identifying poor quality datasets. We thus erred on the side of caution, and tried to avoid eliminating datasets without clear rationale for doing so. We outlined our rationale for discarding any datasets in the S1 Text, with the hope that transparency could suffice in the current absence of more rigorous dataset disqualification criteria.

In addition, we assessed the maximum dataset depth necessary for a study to be appropriately quantified by entropy. We tested several datasets with a range of baseline depths, and performed subsampling to determine a minimum required depth for accurate entropy quantifications **(Figs 3B and S3)**. We defined accuracy based on the deviation from the baseline entropy, and set a threshold of 98% accuracy (corresponding ~0.1 change in entropy). Entropy was relatively robust to subsampling, with 98% accuracy being achieved at above ~2000–4500 counts/cell, depending on the dataset. While this depth was sufficient for most of our assayed datasets, some very low-depth datasets were affected–in particular, all four Drop-seq datasets tested had depths ranging from 1500–4100 counts/cell. Given these results, we omitted the Drop-seq datasets from further analysis.

#### Quality control of poor-quality cells

Outside of dataset filtering, within-dataset quality control is an essential step in all scRNA-seq protocols [41–43]. Protocols will inevitably generate cells that have been lysed or damaged, making them unsuitable for downstream analysis. As our study involves a meta-analysis of many independently generated datasets, we aimed to establish a standardized approach for quality control. This had the dual benefits of ensuring at least a minimal level of comparability while limiting the need to determine individual thresholds for each dataset. We focused on two primary metrics of quality control–cell depth and percentage of reads going to the top 5 highest expressed genes in each cell. We selected these metrics because we observed that they most affected quantification of entropy score **(Fig 3C)** We then defined normalized metrics based on both measurements by dividing the respective measurement by the median of that measure *in that study* and *in that timepoint*. Thus, while comparable cross-study, the metrics could be considered with respect to potential biological and technical variation. We then set a standardized threshold across all studies **(S4 Fig)**.

#### Identifying CMs

In terms of cell-type filtration, our input datasets were fairly heterogeneous, with some including only CMs while others were more broad. Thus, we used SingleCellNet [44] to identify and retain only cells with CM signature. SingleCellNet uses top-scoring pair to enable cross-platform comparisons of test data against a training dataset to annotate celltypes, and has performed well in benchmarking [45]. We used the Tabula Muris [46] as a reference dataset to test against many celltypes. However, as the Tabula Muris is constructed on adult tissues, we were concerned that early-stage CMs may be poorly classified. We thus tested the predicted cell annotations from SingleCellNet across our mouse *in vivo* datasets. We classified a cell as a CM if its score for “cardiac muscle cell” was higher than the score for any other celltype. We found that, while prediction scores for CMs increased over time, CMs were identified as early as e7.5, corresponding appropriately to the onset of cardiomyogenesis **(Fig 3D)**. In human *in vivo* datasets, CMs were present by embryonic week 5, which was the earliest timepoint for which we had data **(S5 Fig)**. These results supported the use of the Tabula Muris reference with SingleCellNet, even for identifying nascent CMs.

### Entropy score enables cross-study inference of maturation status

**Fig 4:**
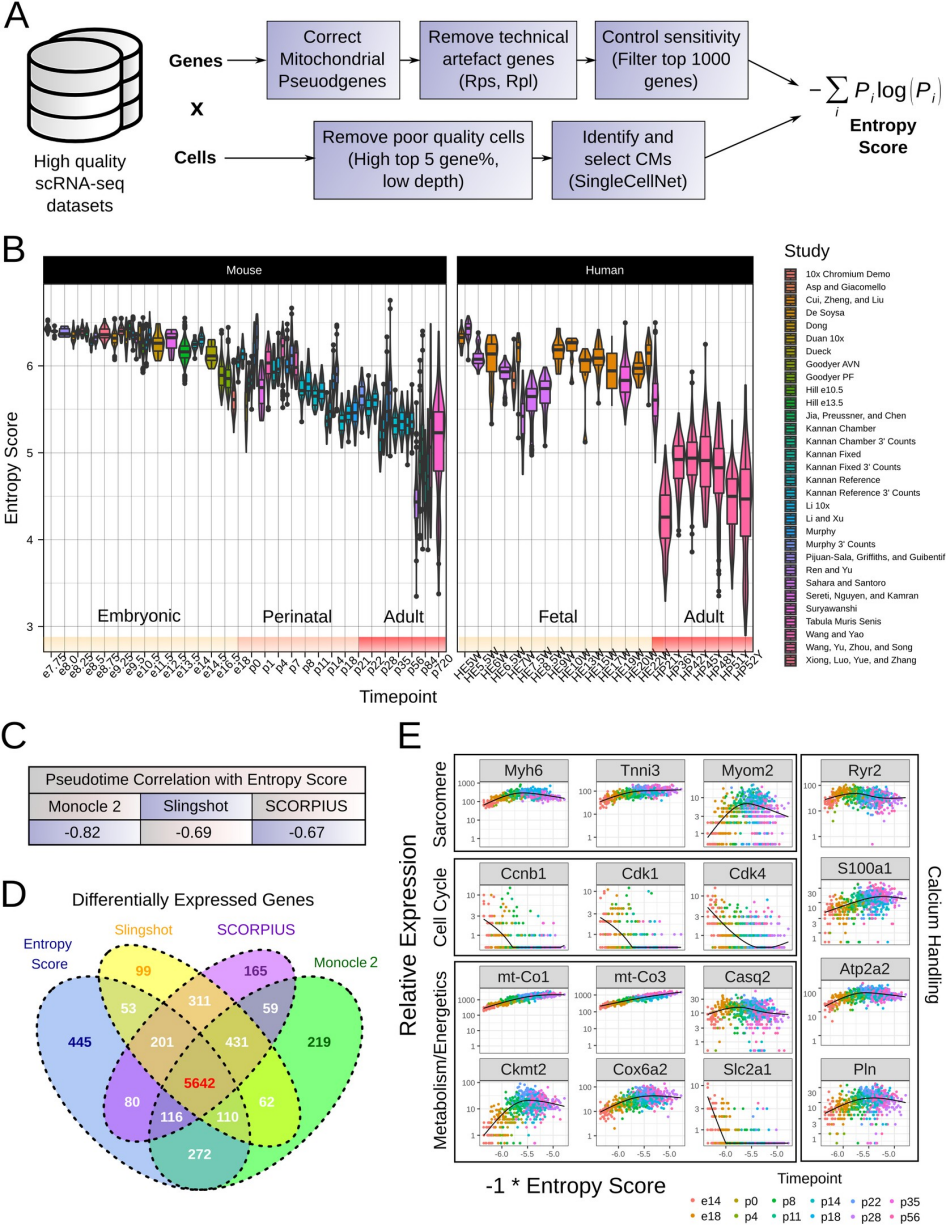
Entropy score enables cross-study and cross-species comparison of CM maturation status, and recapitulates gene trends in CM maturation. **A.** Workflow for computing entropy score from high quality scRNA-seq datasets. **B.** Entropy score for mouse and human in vivo CMs taken from publicly available datasets. HEW = human embryonic week, HPY = human postnatal year. **C.** Pearson correlation between entropy score and calculated pseudotimes for our maturation reference dataset for three trajectory inference methods: Monocle 2, Slingshot, and SCORPIUS. **D.** Venn diagram showing overlap in identified differentially expressed genes between entropy score and trajectory inference methods. Differentially expressed genes were identified by fitting generalized additive models to gene trends over the corresponding pseudotime in Monocle 2, and selecting genes with adjusted p-value < 0.05. **E.** Gene expression trends over entropy score for genes involved in CM maturation, including sarcomeric, cell cycle, metabolism, and calcium handling genes.

Based on the previous results, we developed a workflow for addressing major technical confounding variables to enabling cross-study comparisons **(Fig 4A)**. The output of our workflow is the computed Shannon entropy on the filtered datasets, which we refer to as *entropy score* through the remainder of the manuscript. We tested the utility of entropy score on our previously identified mouse and human *in vivo* CM datasets, which after filtration composed 36,436 CMs. Entropy score gradually decreased over developmental time, as hypothesized by our model **(Figs 4B and S6)**. Notably, despite the marked heterogeneity of dataset characteristics, entropy score was consistent at similar timepoints across multiple datasets. In particular, entropy score showed remarkable concordance between datasets featuring different datatypes. For example, using four UMI-based datasets generated by our group, we found that the ratio of entropy score computed prior to versus after UMI collapsing was 1.02 **(S7 Fig)**.

In the mouse *in vivo* datasets, entropic changes occurred in three broad phases **(Fig 4B)**. In the embryonic phase (~e7.75-e16.5), entropy score decreased at a relatively slow rate. Upon initiation of the perinatal phase at e16.5, entropy score decreased more rapidly before converging onto a relatively mature adult-like phase at p21. These changes correspond well to previous literature about the dynamics of CM maturation, in particular regarding the perinatal CM maturation window [47].

We were additionally curious about the efficacy of entropy score to capture the maturation status of human CMs. We found that there was good concordance in entropy score between stage-matched mouse and human tissues **(Fig 4B)**. In particular, fetal tissues (ranging from embryonic week 5 to embryonic week 22) corresponded approximately to e13.5-e14.5 in mice, while adult human CMs presented with entropy score comparable to adult mouse CMs. We did observe that one dataset (Sahara et al.) showed a notably lower entropy score at embryonic weeks 7–8, though we suspect this may have to do with dataset quality issues. Taken as a whole, however, these results support the use of entropy score as a cross-study, cross-species metric of CM maturation.

### Entropy score recapitulates gene expression trends in CM maturation

We next tested whether entropy score computationally ordered single CMs based on their progression along the maturation process, akin to so-called trajectory inference or pseudotime analysis methods. We selected three well-known trajectory inference methods–Monocle 2, Slingshot, and SCORPIUS–based on their performance in recent benchmarking studies, particularly with reconstructing unidirectional topologies [48]. We then performed trajectory inference with our maturation reference dataset and compared the resultant pseudotimes with entropy score. Additionally, we identified genes differentially expressed over pseudotime/entropy score for each method respectively. Entropy score correlated only moderately with pseudotimes for the three methods **(Figs 4C and S8)**. However, there was notable overlap in identified differentially expressed genes **(Fig 4D)**. In particular, ~93.6% of genes identified as differentially expressed over entropy score were also identified by at least one other method, and ~81.5% were identified as differentially expressed by all methods. Moreover, when treated as a pseudotime metric, entropy score accurately recapitulated known CM maturation gene expression trends **(Fig 4E)**. We further tested entropy score as a pseudotime metric in datasets composed of only one biological timepoint but a range of entropy scores. Intriguingly, gene expression trends across entropy score in these one-timepoint datasets largely matched the trends observed in our maturation reference dataset **(S9 Fig)**. These results suggest that entropy score can effectively reconstruct the CM maturation trajectory as it occurs heterogeneously at the single cell level, and can accurate quantify single CM maturation status regardless of the biological timepoint of the sample.

### Human PSC-CMs do not mature beyond embryonic stage

**Fig 5:**
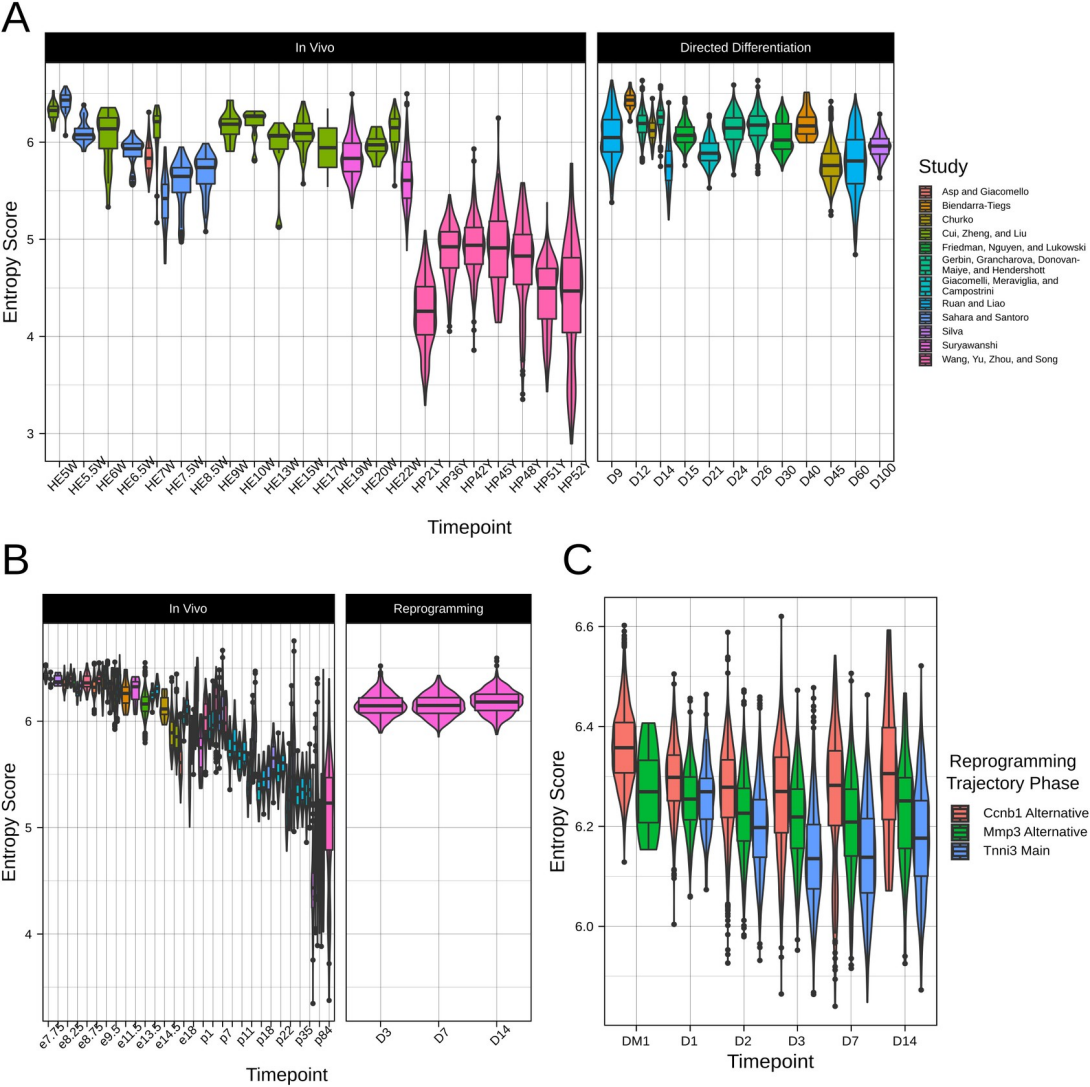
Entropy score quantifies maturation status of PSC-CMs and iCMs. **A**. Comparison in entropy score between human in vivo CMs and human PSC-CMs. Left side of figure reproduced from **Fig 4B**. **B**. Comparison in entropy between mouse in vivo CMs and mouse iCMs. Left side of figure reproduced from **Fig 4B**. **C**. Entropy score for three reprogramming pathways—a canonical Tnnt2+ iCM pathway and two alternative pathways (Ccnb1+ and Mmp3+).

Having validated entropy score as a metric of CM maturation *in vivo*, we next tested the entropy score of PSC-CMs from publicly available datasets **(S2 Table)**. We identified 8 datasets of directed differentiation of human induced PSCs to CMs, and analyzed 13,171 cells between D(ay)9 and D100 of differentiation post-filtering. Though there was some variation from study to study (perhaps due to line-to-line differences or variations in differentiation protocol), there was modest decrease in entropy score over the course of differentiation **(Fig 5A)**. However, no study generated CMs with entropy score lower than human fetal tissues, confirming the immature nature of PSC-CMs. Moreover, there was limited change in entropy score in PSC-CMs after D45, even with long-term culture up to D100, suggestive of maturation arrest. Interestingly, the entropy score of these later timepoint PSC-CMs corresponded to the initiation of the perinatal phase of mouse CM maturation *in vivo*. This observation may point to dysregulation of the endogenous perinatal maturation program during *in vitro* directed differentiation as a cause of poor PSC-CM maturation status, and merits further investigation.

### Reprogrammed CMs present with embryonic-like maturation status

In addition to directed differentiation of PSCs, another approach that has been explored to generate CMs *ex vivo* is direct reprogramming of fibroblasts to CM-like cells (iCMs) by transcription factor, microRNA, and cytokine cocktails [49]. We used entropy score to analyze a dataset of reprogramming of mouse neonatal fibroblasts to iCMs by overexpression of Gata4, Mef2c, and Tbx5. Focusing only on cells with CM-like signature, we found that entropy score showed limited change between D3 and D14 of reprogramming **(Fig 5B)**. iCMs remained at a mid-embryonic stage of maturation, comparable to e13.5-e14.5 in mouse *in vivo* CMs. Moreover, compared to PSC-CMs at the same timepoint of differentiation, iCMs displayed higher entropy. This result matches earlier findings that direct reprogramming less effectively recapitulates native gene regulatory networks compared to directed differentiation [50].

We further explored change in entropy score across multiple reprogramming pathways. The authors of the dataset identified a branching reprogramming trajectory [51]. Reprogrammed cells entered either a canonical iCM route (e.g. Tnni3^+^) or two alternative pathways–one characterised by activation of Mmp3 and another marked by cell cycle progression (e.g. Ccnb1^+^). Using the authors’ annotations, we classified all cells in the dataset (including those without a CM signature) into one of these three pathways and assessed the entropy score for cells in each pathway **(Fig 5C)**. At D1 of reprogramming, cells in all three pathways show similar entropy score. However, from D1 to D3, cells in the canonical iCM pathway show more notable decrease in entropy score, and indeed remain at a lower entropy score than cells in other pathways. Thus, while iCMs still present with a notably immature status compared to *in vivo*, they display some improvement in maturation status compared to cells arrested in alternative reprogramming pathways.

### Entropy score decreases over pancreatic beta cell and hepatocyte maturation

**Fig 6:**
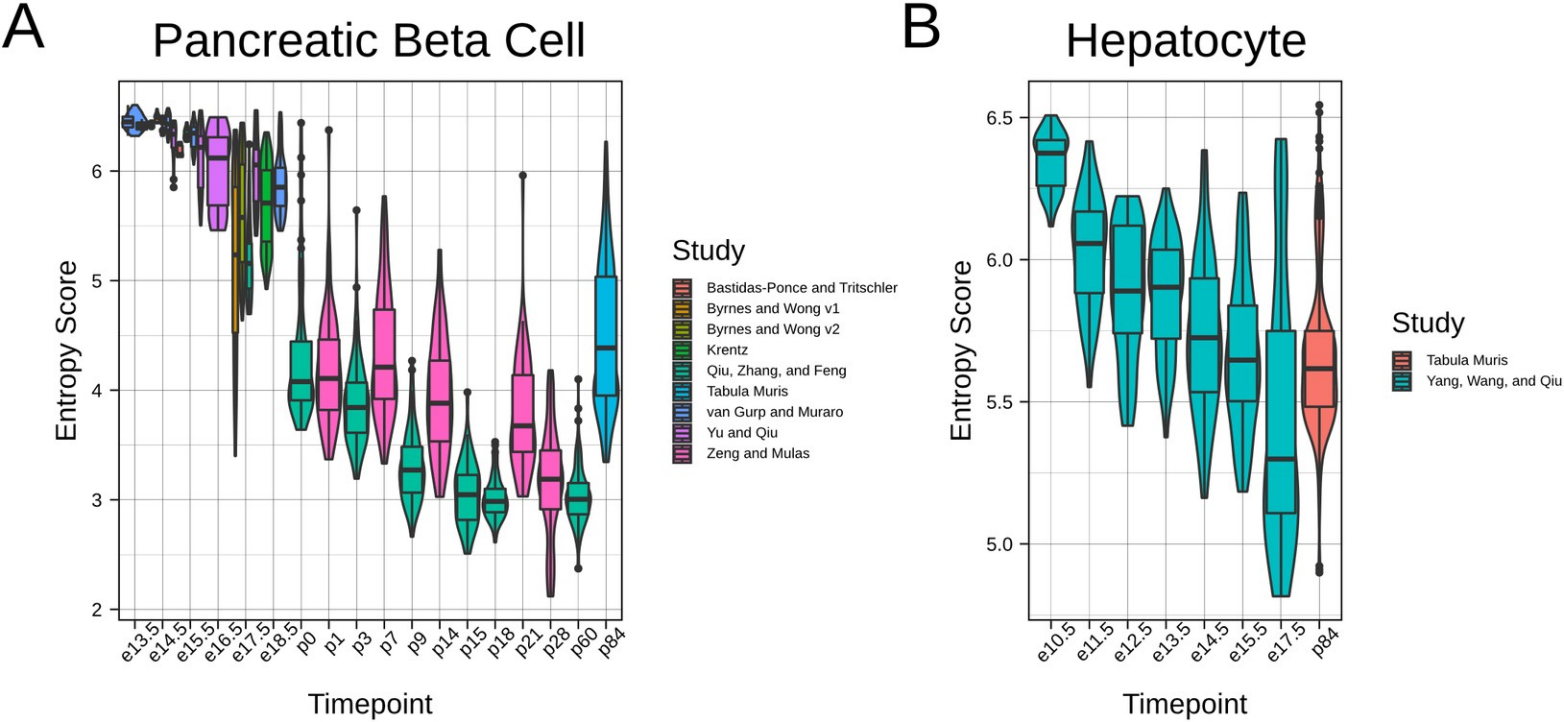
Entropy score decreases over maturation in non-CM tissue contexts. **A.** Entropy score for mouse in vivo pancreatic beta cells taking from publicly available datasets. **B.** Entropy scores for mouse in vivo hepatocytes.

We primarily focused our attention to the challenge of quantifying PSC-CM maturation, given the significant clinical need for generating CMs *ex vivo*. However, incomplete maturation and difficulty in assessing maturation status affect other tissue contexts as well. As proof of concept, we computed entropy score for *in vivo* mouse datasets of pancreatic beta cells **(Fig 6A)** and hepatocytes **(Fig 6B)**. As with CMs, entropy score decreased over time for both celltypes, though with celltype-specific dynamics. For example, beta cells show a large postnatal drop in entropy score, likely corresponding to birth-related metabolic changes and need for insulin. By contrast, hepatocytes show a more steady decline in entropy score, though further datasets will be necessary to more thoroughly characterize these dynamics. Moreover, it must be noted that unlike CMs, beta cells and hepatocytes may continue to proliferate postnatally [52,53], which may affect the interpretation of the entropy score in older tissues. Nevertheless, these preliminary results support the applicability of entropy score to non-cardiac tissue engineering contexts as well.

## Discussion

Here, we present the use of transcriptomic entropy score for quantifying cellular maturation at the single cell level. Our approach builds on the well-known Shannon entropy to generate a metric of CM maturation from scRNA-seq data that is robust to a range of sequencing protocols and potential batch effects. In particular, entropy score enables direct benchmarking of *in vitro* PSC-CM maturation against their *in vivo* counterparts. This is particularly important because endogenous development is the gold standard for instructing PSC-derived tissue maturation. Correspondingly, we believe that perturbations to engineer maturation *must* be compared against this gold standard rather than an *in vitro* control. Our newly developed entropy score enables comparison of PSC-CMs against the full trajectory of endogenous CM maturation. Entropy score can thus be used to better assess PSC-CM maturation methodologies, and guide development of tissues that better recapitulate the adult CM phenotype. Moreover, while we focused on CM maturation here, we demonstrated the extensibility to other tissues as well. Given the increasing availability of both endogenous and PSC-derived scRNA-seq datasets, we expect that broad application of entropy score will enable development of improved tissues for clinical use.

It should be noted, however, that we do not see entropy score as the end-all for maturation quantification. In addition to potential discrepancies between transcript and protein level expression [54], the mature cellular phenotype encompasses numerous functional parameters that may be only partially captured at the transcriptomic level [55]. We envision entropy score as complementing existing celltype-specific functional assays to advance a more complete assessment of single cell maturation status. One other caveat that must be noted is that similar entropy score alone does not guarantee identical or matching gene expression patterns between samples. However, our results do indicate that entropy score serves as a strong general readout of tissue maturation. Moreover, given the difficulty of comparing expression values across scRNA-seq datasets, entropy score may serve as a useful approximation of identifying samples with similar maturation states.

Through meta-analysis of over 45 scRNA-seq datasets of CMs, we were able to gain some insights into the dynamics of CM maturation. In particular, we were interested to note the existence of a perinatal phase of maturation *in vivo*, initiating at approximately e16.5-e18.5, during which CM entropy score rapidly decreased. Entropy score continued to decrease until approximately ~3–4 weeks postnatally. We previously hypothesized the existence of a critical perinatal window for CM maturation, and postulated that disruption of this window *in vitro* leads to maturation arrest [47]. The significant decrease in entropy observed in our study supports the perinatal window hypothesis. Moreover, late-stage PSC-CMs remained arrested at an entropy score similar to those of e16.5 CMs *in vivo*. To date, mechanistic understanding of PSC-CM maturation arrest has been limited, but may involve progressive disruption of cardiac gene regulatory networks [17]. Our results suggest that increased focus should be placed on trying to understand regulators of perinatal maturation *in vivo*, and determining discrepancies in activity of these regulators *in vitro*. In particular, we anticipate that entropy score can serve as a readout for mosaic-based screening systems for identifying perinatal CM maturation regulators [56]. Whether similar mechanisms underlie maturation arrest in other PSC-derived cells remains a question for further studies.

In this study, we found that entropy score could be applied to scRNA-seq datasets generated from a wide range of protocols. Excluding the quality control steps, entropy score is computed from information in one cell at a time, independent of other cells or datasets. Nevertheless, entropy score shows strong concordance with CM maturation status in a comparable manner across dataset. This is particular novel as, thus far, direct comparisons across studies has been limited by confounding batch effects. Moreover, current batch correction algorithms may be poorly suited to integration of datasets along a continuous trajectory. Additionally, scaling batch correction algorithms to many datasets may be complex and computationally intensive. By contrast, entropy score has limited computational demands and can scale easily to allow for comparison of many datasets.

We were particularly intrigued to note the comparability of entropy scores across datasets with entirely different datatypes (e.g. reads, UMIs). For example, it is well known that PCR amplification in scRNA-seq protocols can lead to biases [57], which was one of the motivations for the development of UMIs. However, entropy scores were comparable for UMI datasets prior to and after collapsing UMIs. Likewise, datasets generated from full-length protocols did not display notable biases in entropy score. This observation may have been incidental to the datasets we studied–for example, high quality datasets may have presented with sufficiently low amplification bias to enable comparison. It is possible that entropy score is less robust to more extreme cases of amplification bias. We do not believe our finding precludes the use of best practices for scRNA-seq protocols, including the use of UMIs for many experimental designs. Nevertheless, we were encouraged that entropy score could be used to facilitate cross-comparison between otherwise incompatible datatypes.

One technical limitation of entropy score was its poor performance with Drop-seq datasets. We consistently found that Drop-seq datasets presented with higher entropy than data generated at similar timepoints through other protocols. This may be a consequence of depth; the Drop-seq datasets that we tested were the lowest depth studies tested and below our identified optimal depth threshold. However, given the increasing prevalence of other high-quality droplet-based protocols (in particular, 10x Chromium), we believe this is not a major limiting factor to the use of entropy score. We additionally did not test single nuclear RNA-seq datasets, both due to concerns of depth and because we expected that the gene distribution would be inherently different from whole cell studies [58]. Nevertheless, the emergence of methods for isolation of whole adult CMs in mouse and human [32,59,60] may reduce the future need for nuclear RNA-seq.

At the single cell level, CM maturation proceeds heterogeneously along a unidirectional trajectory [33]. We were therefore curious to know the extent to which entropy score could capture single cell positioning along this trajectory, in effect functioning as a pseudotime metric. Entropy score only modestly correlated with other established pseudotime methods, though all methods recovered similar differentially expressed genes. These discrepancies may be due to transcriptomic noise in single cell data. However, it should be emphasized that entropy score works in a fundamentally different manner than many trajectory inference methods. Most trajectory inference methods utilize some type of dimensionality reduction step prior to curve fitting. By contrast, outside of the subselection of highly expressed genes, entropy score uses no dimensionality reduction step. Moreover, entropy score makes no assumptions about relationships between cells–all relevant information is calculated independently for each cell. Despite being agnostic to cell-cell relationships, entropy score accurately captures CM maturation expression trends. Commonly used dimensionality reduction methods have been shown to distort local neighbourhoods and affect trajectory reconstruction [61], and thus entropy score may more optimally capture single CM dynamics in maturation.

Entropy score has several important antecedents that must be acknowledged. Our work is similar to StemID [29], which uses Shannon entropy to assign progenitor state within a trajectory. We extend this usage with several gene filtering steps to better facilitate cross-study comparison. Shannon entropy is also utilized in SLICE [27], which computes entropy based on functional annotations of genes, and SCENT [25], which computes entropy within a protein-protein interaction network. Both approaches are powerful for constructing trajectories for differentiating cells. However, unlike differentiation, CM maturation is characterized by continuous rather than step-wise or switch-like changes. For this purpose, an entropy score built directly on gene expression levels is both simpler to compute and more appropriate. Lastly, our work is similar conceptually to CytoTRACE [28], which leverages gene diversity to order cells by differentiation status. Directly comparing number of genes expressed by each cell is confounded by cross-study differences in depth and sensitivity, however. CytoTRACE addresses this by using a smoothing step within dataset. However, this limits its use for datasets with few cells or representing fewer maturation states. By contrast, outside of quality filtering, entropy score performs computations on each cell independently, extending its utility to more datasets. We believe these differences improve the utility of entropy score for benchmarking the maturation status of PSC-derived tissues.

## Methods

### Ethics statement

All animal experiments done as part of this manuscript were approved under protocols by the Johns Hopkins Animal Care and Use Committee (IACUC Welfare Assurance number A3271-01).

Raw data for the maturation reference can be found on GEO at GSE147807. Code to generate figures in this manuscript as well as the counts tables for the datasets analyzed in this manuscript can be found on Github at https://github.com/skannan4/cm-entropy-score.

Shannon entropy has had long-standing applications in developmental biology as well as transcriptional analysis [30,62]. A standard form for Shannon entropy *S* is:

$$S=-\sum_{i}P_{i}\;log(P_{i})$$

where *P*_*i*_ represents individual probabilities for events of interest. Here, we define *P*_*i*_ as the probability of selecting a given gene *i* in a cell. From scRNA-seq data, this can be computed by simply dividing the number of counts for gene *i* by all of the gene counts in a given cell. For our entropy score, we similarly use Shannon entropy, except after subsetting the top 1000 highest expressed genes to enable sensitivity control.

### Generation of maturation reference

#### Mice

To generate mice for our reference dataset, we crossed B6.FVB-Tg(Myh6-cre)2182Mds/J mice (aMHC-cre, Jackson Laboratory, Stock No. 011038) with B6.129(Cg)-Gt(ROSA)26Sor^tm4(ACTB-tdTomato,-EGFP)Luo/J^ (mTmG, Jackson Laboratory, Stock No. 007676). Both mice have C57BL/6J congenic background. All animals were maintained compliant to protocols by the Johns Hopkins Animal Care and Use Committee.

#### CM isolation

For isolation of CMs from e14-p4 timepoints, we used the neonatal cardiomyocyte isolation kit from Miltenyi Biotec in conjunction with the gentleMACS Dissociator. For later timepoints, we performed Langendorff isolation of CMs. We prepared the following buffers:

- Perfusion buffer: 120 mM NaCl, 5.4 mM KCl, 1.2 mM NaH_2_PO_4_, 20 mM NaHCO_3_, 5.5 mM glucose, 5 mM BDM, 5 mM Taurine, and 1 mM MgCl_2_, adjusted to pH 7.4
- Digestion buffer: 40 mL Perfusion buffer plus 35.8 mg Collagenase Type II (Worthington CLS-2), 3 mg Protease (Sigma P5147)
- Tyrode’s buffer: 140 mM NaCl, 5 mM KCl, 10 mM HEPES, 5.5 mM glucose, and 1 mM MgCl_2_, adjusted to pH 7.4

We used a horizontal (i.e. non-hanging) Langendorff apparatus with a chamber filled with perfusion buffer. To perform isolation, we first performed isofluorane anaesthesia on non-heparinized mice. Mice were observed until clearly anaesthetized and unresponsive to toe pinch, and subsequently euthanized by cervical dislocation. The heart was then rapidly excised from the chest and cannulated to the Langendorff apparatus. Flow time and rate of flow were dependent on the age of the mouse and were typically judged based on completeness of digestion to touch. Subsequently, the left ventricular free wall was excised and minced. We filtered isolated cells through a 100 μM screen to eliminate large tissue chunks, spun down at 800 RPM for 1 minute (Eppendorf centrifuge 5702), and resuspended cells in 10 mL Tyrode’s buffer.

#### LP-FACS

We have detailed our LP-FACS approach previously [32]. We reproduce our methods here. We utilized a COPAS SELECT instrument (Union Biometrica). The COPAS SELECT was updated and rebranded as the FP-500, but the protocol here study does not use the new features and thus the two are functionally indistinguishable. We optimized sorting for cardiomyocytes by using a sort delay of 8 and sort width of 6. Additionally, we used the following fluorescence settings: ext gain 50, green gain 200, yellow gain 200, red gain 255, extension integral gain 50, green integral gain 200, yellow integral gain 200, red integral gain 255, green PMT 800, yellow PMT 800, red PMT 1100. Coincidence check was selected to ensure proper single event sorting. We typically flowed cells between 20–60 events/second. We maintained cells in Tyrode’s buffer during the sort and sorted them into Tyrode’s buffer. To run the machine, we used ClearSort Sheath Fluid (Sony, Lot 1218L345).

#### scRNA-seq library preparation and sequencing

We performed sequencing using the mcSCRB-seq protocol [63]. The protocol has been described at protocols.io at dx.doi.org/10.17504/protocols.io.p9kdr4w. Pooled libraries were sequenced on one mid-output lanes of the Illumina NextSeq500 with 16 base pair barcode read, 8 base pair i7 index read, and 66 base pair cDNA read design.

### Computational analyses

All analyses performed in the paper were done in R; code to reproduce the figures can be found at our Github (https://github.com/skannan4/cm-entropy-score). Dataset characteristics are presented in **S1 and S2 Tables**, and details of each individual dataset are described in the S1 Text. Dimensionality reduction as well as dataset integration for **Fig 1** was done using Monocle 3. Differential gene expression analysis for **Fig 5** was done using Monocle 2, replacing Monocle 2’s generated pseudotime with entropy score or pseudotime from other methods as appropriate.

## Supporting information

S1 Fig Correction of mitochondrial pseudogenes enables consistent entropy score measurements across mapping/counting pipelines. **A.** Entropy scores for the maturation reference dataset mapped by zUMIs, kallisto|bustools with the full reference, and kallisto|bustools with the CellRanger reference. Pre- and post-correction scores are shown. **B.** As in **A**, showing mitochondrial proportions. **C.** Entropy scores for the 10x Chromium heart dataset mapped by zUMIs, kallisto|bustools with the full reference, kallisto|bustools with the CellRanger reference, and CellRanger. **D.** As in **C**, showing mitochondrial proportions.
(TIFF)

S2 Fig Ribosomal protein-coding genes are expressed in a sequencing protocol-specific manner. **A.** Proportion of ribosomal protein coding genes in mouse *in vivo* datasets, grouped by timepoint. **B.** Proportion of ribosomal protein coding genes in mouse *in vivo* datasets, grouped by library preparation method. 10x v1-v3 protocols have been coalesced together for the purposes of this figure.
(TIFF)

S3 Fig Entropy score is robust across a range of sequencing depths. For each of four datasets, we performed subsampling and computed the entropy score as well as accuracy (calculated as deviation from baseline entropy score). At each stage, we included only cells with genes > 1000, and subsampled only to a depth where the median number of genes remained > 1000. Data is shown for **A**-**B**. Dueck et al. **C**-**D**. Jia et al. at e9.5. **E**-**F**. First 100 cells from Hill et al. at e10.5. **G**-**H**. First 100 cells from Duan et al.
(TIFF)

S4 Fig Poor quality single cells can be identified and removed with normalized depth and top 5 gene percentage metrics. **A.** Normalized depth QC metric for all datasets. Red line indicates the threshold of −0.5. **B.** Normalized top 5 gene percentage metric for all datasets. Red line indicates the threshold of 1.3.
(TIFF)

S5 Fig SingleCellNet identifies single cells with CM signature. Cells are labeled based on whether their highest classification was for “cardiac muscle” or another celltype. **A.** For human *in vivo* datasets. **B.** For human *in vitro* directed differentiation datasets.
(TIFF)

S6 Fig Entropy score enables comparison of maturation status of CMs from scRNA-seq datasets with diverse characteristics. This figure corresponds to **Fig 2B,** but with boxplots coloured by **A.** sequencing protocol and **B.** isolation method.
(TIFF)

S7 Fig Entropy score is consistent for UMI datasets pre- and post-UMI collapsing. **A.** Ratio of entropy score for UMI datasets computed prior to vs. after UMI collapsing.
(TIFF)

S8 Fig Entropy score correlates modestly with previous trajectory inference methods. We reconstructed trajectories of our maturation reference dataset using **A-B**. Monocle 2, **C-D**. Slingshot, and **E-F**. SCORPIUS.
(TIFF)

S9 Fig Entropy score captures CM maturation-related gene expression trends in one-timepoint datasets. Gene trends across entropy score, as in **Fig 3C**, are plotted for **A.** 10x Chromium heart dataset, **B.** Goodyer et al., and **C.** Duan et al.
(TIFF)

S1 Table In vivo datasets used for this study (TIFF)

S2 Table PSC-CM and iCM datasets used for this study (TIFF)

S1 Text Appendix for all datasets analyzed in this study (DOCX)

## References

[1] Elitt MS, Barbar L, Tesar PJ. Drug screening for human genetic diseases using iPSC models. Hum Mol Genet. 2018;27(May):89–98. doi: 10.1093/hmg/ddy186 29771306 PMC6061782

[2] Ebert AD, Liang P, Wu JC. Induced Pluripotent Stem Cells as a Disease Modeling and Drug Screening Platform. J Cardiovasc Pharmacol. 2012;60(4):408–16. doi: 10.1097/FJC.0b013e318247f642 22240913 PMC3343213

[3] Rowe RG, Daley GQ. Induced pluripotent stem cells in disease modelling and drug discovery. Nat Rev Genet [Internet]. 2019;20(July). Available from: doi: 10.1038/s41576-019-0100-z 30737492 PMC6584039

[4] Nguyen HT, Jacobs K, Spits C. Human pluripotent stem cells in regenerative medicine: where do we stand? Reproduction. 2015; 10.1530/REP-18-0291 30325181

[5] Hirschi KK, Li S, Roy K. Induced Pluripotent Stem Cells for Regenerative Medicine. Annu Rev Biomed Eng. 2014; doi: 10.1146/annurev-bioeng-071813-105108 24905879 PMC4287204

[6] Feric NT, Radisic M. Maturing human pluripotent stem cell-derived cardiomyocytes in human engineered cardiac tissues ☆. Adv Drug Deliv Rev [Internet]. 2016;96:110–34. Available from: doi: 10.1016/j.addr.2015.04.019 25956564 PMC4635107

[7] Corbett JL, Duncan SA. iPSC-Derived Hepatocytes as a Platform for Disease Modeling and Drug Discovery. Front Med. 2019;6(November):1–12. doi: 10.3389/fmed.2019.00265 31803747 PMC6873655

[8] Shahjalal H, Dayem AA, Lim KM, Jeon T, Cho S. Generation of pancreatic β cells for treatment of diabetes: advances and challenges. Stem Cell Res Ther. 2018;3. doi: 10.1186/s13287-018-1099-3 30594258 PMC6310974

[9] Wu Y, Chiu F, Yeh C, Kuo H. Opportunities and challenges for the use of induced pluripotent stem cells in modelling neurodegenerative disease. Open Biol. 2019;9. doi: 10.1098/rsob.180177 30958120 PMC6367134

[10] Zhu R, Blazeski A, Poon E, Costa KD, Tung L, Boheler KR. Physical developmental cues for the maturation of human pluripotent stem cell-derived cardiomyocytes. Stem Cell Res Ther. 2014;5:1–18. doi: 10.1186/scrt390 25688759 PMC4396914

[11] Nguyen AH, Marsh P, Schmiess-heine L, Burke PJ, Lee A, Lee J. Cardiac tissue engineering: state-of-the-art methods and outlook. J Biol Eng. 2019;13:1–21. doi: 10.1186/s13036-018-0125-4 31297148 PMC6599291

[12] Scuderi GJ, Butcher J. Naturally Engineered Maturation of Cardiomyocytes. Front Cell Dev Biol [Internet]. 2017;5(May):1–28. Available from: http://journal.frontiersin.org/article/10.3389/fcell.2017.00050/full 28529939 10.3389/fcell.2017.00050 PMC5418234

[13] Chen C, Soto-gutierrez A, Baptista PM, Spee B. Biotechnology Challenges to In Vitro Maturation of Hepatic Stem Cells. Gastroenterology [Internet]. 2018;154(5):1258–72. Available from: doi: 10.1053/j.gastro.2018.01.066 29428334 PMC6237283

[14] B L-V, Rashidi H, Cameron K, Hay DC. Pluripotent stem cell derived hepatocytes: using materials to define cellular differentiation and tissue engineering. J Mater Chem B. 2016;3433–42. doi: 10.1039/c6tb00331a 27746914 PMC5024673

[15] Bertucci TB, Dai G. Biomaterial Engineering for Controlling Pluripotent Stem Cell Fate. Stem Cells Int. 2018;2018. doi: 10.1155/2018/9068203 30627175 PMC6304878

[16] Cai W, Zhang J, Lange WJ De, Gregorich ZR, Karp H, Farrell ET, et al. An Unbiased Proteomics Method to Assess the Maturation of Human Pluripotent Stem Cell-Derived Cardiomyocytes. Circ Res. 2019;125:936–53. doi: 10.1161/CIRCRESAHA.119.315305 31573406 PMC6852699

[17] Uosaki H, Cahan P, Lee DI, Wang S, Miyamoto M, Fernandez L, et al. Transcriptional Landscape of Cardiomyocyte Maturation. Cell Rep [Internet]. 2015;13(8):1705–16. Available from: doi: 10.1016/j.celrep.2015.10.032 26586429 PMC4662925

[18] van den Berg CW, Okawa S, Chuva de Sousa Lopes SM, van Iperen L, Passier R, Braam SR, et al. Transcriptome of human foetal heart compared with cardiomyocytes from pluripotent stem cells. Development [Internet]. 2015;142(18):3231–8. Available from: http://dev.biologists.org/cgi/doi/10.1242/dev.123810 26209647 10.1242/dev.123810

[19] Kim D, Ryu J, Son M, Oh J, Chung K, Lee S, et al. A Liver-Specific Gene Expression Panel Predicts the Differentiation Status of In Vitro Hepatocyte Models. Hepatology. 2017;66(5):1662–74. doi: 10.1002/hep.29324 28640507 PMC5698781

[20] Stegle O, Teichmann SA, Marioni JC. Computational and analytical challenges in single-cell transcriptomics. Nat Rev Genet. 2015;16(January). doi: 10.1038/nrg3833 25628217

[21] Tung P, Blischak JD, Hsiao CJ, Knowles DA, Burnett JE, Pritchard JK, et al. Batch effects and the effective design of single-cell gene expression studies. Sci Rep [Internet]. 2017;7(September 2016):1–15. Available from: doi: 10.1038/s41598-016-0028-x 28045081 PMC5206706

[22] Tran HTN, Ang KS, Chevrier M, Zhang X, Yee NSL, Goh M, et al. A benchmark of batch-effect correction methods for single-cell RNA sequencing data. Genome Biol. 2020;21(12):1–32. doi: 10.1186/s13059-019-1850-9 31948481 PMC6964114

[23] Benjamin et al. EJ. Heart Disease and Stroke Statistics—2018 Update A Report From the American Heart Association. 2018. 67–492 p. 10.1161/CIR.0000000000000558 29386200

[24] Teschendorff AE, Sollich P, Kuehn R. Signalling entropy: A novel network-theoretical framework for systems analysis and interpretation of functional omic data. Methods [Internet]. 2014;67(3):282–93. Available from: doi: 10.1016/j.ymeth.2014.03.013 24675401

[25] Teschendorff AE, Enver T. Single-cell entropy for accurate estimation of differentiation potency from a cell’s transcriptome. Nat Commun [Internet]. 2017;8:1–15. Available from: doi: 10.1038/s41467-016-0009-6 28569836 PMC5461595

[26] Chen W, Teschendorff AE. Estimating Differentiation Potency of Single Cells Using Single-Cell Entropy (SCENT). Comput Methods Single-Cell Data Anal. 2019;1935:125–39. doi: 10.1007/978-1-4939-9057-3_9 30758824

[27] Guo M, Bao EL, Wagner M, Whitsett JA, Xu Y. SLICE: determining cell differentiation and lineage based on single cell entropy. Nucleic Acids Res. 2017;45(7):1–14. doi: 10.1093/nar/gkw1278 27998929 PMC5397210

[28] Gulati GS, Sikandar SS, Wesche DJ, Manjunath A, Berger MJ, Ilagan F, et al. Single-cell transcriptional diversity is a hallmark of developmental potential. Science (80-). 2020;367:405–11. doi: 10.1126/science.aax0249 31974247 PMC7694873

[29] Grun D, Muraro MJ, Boisset J-C, Wiebrands K, Lyubimova A, Dharmadhikari G, et al. De Novo Prediction of Stem Cell Identity using Resource De Novo Prediction of Stem Cell Identity using Single-Cell Transcriptome Data. Cell Stem Cell. 2016;19:266–77. doi: 10.1016/j.stem.2016.05.010 27345837 PMC4985539

[30] Macarthur BD, Lemischka IR. Statistical Mechanics of Pluripotency. Cell [Internet]. 2013;154(3):484–9. Available from: doi: 10.1016/j.cell.2013.07.024 23911316

[31] Ackers-johnson M, Lek W, Tan W, Foo RS. Following hearts, one cell at a time: recent applications of single-cell RNA sequencing to the understanding of heart disease. Nat Commun [Internet]. 2018;8–11. Available from: doi: 10.1038/s41467-017-01586-1 30375391 PMC6207674

[32] Kannan S, Miyamoto M, Lin BL, Zhu R, Murphy S, Kass D, et al. Large particle fluorescence-activated cell sorting enables high-quality single-cell RNA sequencing and functional analysis of adult cardiomyocytes. Circ Res. 2019; doi: 10.1161/CIRCRESAHA.119.315493 31415233 PMC6699769

[33] Murphy S, Miyamoto M, Kervadec A, Kannan S, Tampakakis E, Lin BL, et al. PGC1/PPAR drive cardiomyocyte maturation at single cell level via Yap1 and SF3B2. Nat Commun [Internet]. 2021;12(1):1648. Available from: doi: 10.1038/s41467-021-21957-z 33712605 PMC7955035

[34] DeLaughter DM, Bick AG, Wakimoto H, McKean D, Gorham JM, Kathiriya IS, et al. Single-Cell Resolution of Temporal Gene Expression during Heart Development. Dev Cell [Internet]. 2016;39(4):480–90. Available from: doi: 10.1016/j.devcel.2016.10.001 27840107 PMC5198784

[35] Wang Y, Yao F, Wang L, Li Z, Ren Z, Li D, et al. Single-cell analysis of murine fibroblasts identifies neonatal to adult switching that regulates cardiomyocyte maturation. Nat Commun [Internet]. 2020;11(1). Available from: doi: 10.1038/s41467-020-16204-w 32444791 PMC7244751

[36] Haghverdi L, Lun ATL, Morgan MD, Marioni JC. Batch effects in single-cell RNA-sequencing data are corrected by matching mutual nearest neighbors. Nat Biotechnol. 2018;36(5). doi: 10.1038/nbt.4091 29608177 PMC6152897

[37] Malik AN, Czajka A, Cunningham P. Accurate quantification of mouse mitochondrial DNA without co-amplification of nuclear mitochondrial insertion sequences. Mitochondrion. 2016;29:59–64. doi: 10.1016/j.mito.2016.05.003 27181048

[38] Parekh S, Ziegenhain C, Vieth B, Enard W, Hellmann I, Str G. zUMIs—A fast and flexible pipeline to process RNA sequencing data with UMIs. 2018;(May):1–9. 10.1093/gigascience/giy059 PMC6007394 29846586

[39] Melsted P, Booeshaghi AS, Gao F, Beltrame E, Lu L, Hjorleifsson KE, et al. Modular and efficient pre-processing of single-cell RNA-seq. bioRxiv. 2019;1–18.

[40] Ziegenhain C, Vieth B, Parekh S, Reinius B, Guillaumet-Adkins A, Smets M, et al. Comparative Analysis of Single-Cell RNA Sequencing Methods. Mol Cell. 2017;631–43. doi: 10.1016/j.molcel.2017.01.023 28212749

[41] Ilicic T, Kim JK, Kolodziejczyk AA, Bagger FO, Mccarthy DJ, Marioni JC, et al. Classification of low quality cells from single-cell RNA-seq data. Genome Biol [Internet]. 2016;17:1–15. Available from: doi: 10.1186/s13059-015-0866-z 26887813 PMC4758103

[42] Luecken MD, Theis FJ. Current best practices in single-cell RNA-seq analysis: a tutorial. Mol Syst Biol. 2019;15. doi: 10.15252/msb.20188746 31217225 PMC6582955

[43] Mccarthy DJ, Campbell KR, Lun ATL, Wills QF. Scater: pre-processing, quality control, normalization and visualization of single-cell RNA-seq data in R. Bioinformatics. 2017;33(January):1179–86. doi: 10.1093/bioinformatics/btw777 28088763 PMC5408845

[44] Tan Y, Cahan P. SingleCellNet: a computational tool to classify single cell RNA-Seq data across platforms and across species. bioRxiv. 2018; 10.1016/j.cels.2019.06.004 PMC6715530 31377170

[45] Abdelaal T, Michielsen L, Cats D, Hoogduin D, Mei H, Reinders MJT, et al. A comparison of automatic cell identification methods for single-cell RNA sequencing data. Genome Biol. 2019;20:1–19. doi: 10.1186/s13059-018-1612-0 31500660 PMC6734286

[46] Consortium TM. Single-cell transcriptomics of 20 mouse organs creates a Tabula Muris. Nature. 2018;562. doi: 10.1038/s41586-018-0590-4 30283141 PMC6642641

[47] Kannan S, Kwon C. Regulation of cardiomyocyte maturation during critical perinatal window. J Physiol. 2019;0(June 2018):1–16. doi: 10.1113/JP276754 30571853 PMC7682257

[48] Saelens W, Cannoodt R, Todorov H, Saeys Y. A comparison of single-cell trajectory inference methods. Nat Biotechnol [Internet]. 2019;37(May). Available from: doi: 10.1038/s41587-019-0071-9 30936559

[49] Tani H, Sadahiro T, Ieda M. Direct Cardiac Reprogramming: A Novel Approach for Heart Regeneration. Int J Mol Sci. 2018;19:1–13. doi: 10.3390/ijms19092629 30189626 PMC6165160

[50] Cahan P, Li H, Morris SA, Lummertz E, Daley GQ. CellNet: Network Biology Applied to Stem Cell Engineering. Cell. 2014;158:903–15. doi: 10.1016/j.cell.2014.07.020 25126793 PMC4233680

[51] Stone NR, Gifford CA, Thomas R, Ivey KN, Pollard KS, Stone NR, et al. Context-Specific Transcription Factor Functions Regulate Epigenomic and Transcriptional Dynamics during Cardiac Reprogramming. Cell Stem Cell [Internet]. 2019;25(1):87–102.e9. Available from: doi: 10.1016/j.stem.2019.06.012 31271750 PMC6632093

[52] Bonner-Weir S, Aguayo-Mazzucato C, Weir GC. Dynamic development of the pancreas from birth to adulthood. Ups J Med Sci. 2016;121(2):155–8. doi: 10.3109/03009734.2016.1154906 26998806 PMC4900072

[53] Miyaoka Y, Miyajima A. To divide or not to divide: Revisiting liver regeneration. Cell Div. 2013;8(1):1–12. doi: 10.1186/1747-1028-8-1 23786799 PMC3695844

[54] Blevins WR, Tavella T, Moro SG, Blasco-moreno B, Closa-mosquera A, Díez J, et al. Extensive post-transcriptional buffering of gene expression in the response to severe oxidative stress in baker’s yeast. Sci Rep. 2019;9(July):1–11. doi: 10.1038/s41598-019-47424-w 31358845 PMC6662803

[55] Gerbin KA, Grancharova T, Donovan-maiye R, Hendershott MC, Dinh SQ, Gehring JL, et al. Cell states beyond transcriptomics: integrating structural organization and gene expression in hiPSC-derived cardiomyocytes. bioRxiv. 2020; 10.1016/j.cels.2021.05.001 34043964

[56] VanDusen NJ, Lee JY, Gu W, Sethi I, Zheng Y, King JS, et al. In vivo CRISPR screening identifies RNF20/40 as epigenetic regulators of cardiomyocyte maturation. bioRxiv. 2019; 10.1038/s41467-021-24743-z PMC8295283 34290256

[57] Islam S, Zeisel A, Joost S, Manno G La, Zajac P, Kasper M, et al. Quantitative single-cell RNA-seq with unique molecular identifiers. Nat Methods. 2014;11(1). doi: 10.1038/nmeth.2772 24363023

[58] Selewa A, Dohn R, Eckart H, Lozano S, Xie B, Gauchat E, et al. Systematic Comparison of High- throughput Single-Cell and Single- Nucleus Transcriptomes during Cardiomyocyte Differentiation. Sci Rep. 2020;10:1–13. doi: 10.1038/s41598-019-56847-4 32001747 PMC6992778

[59] Yekelchyk M, Guenther S, Preussner J, Braun T. Mono—and multi—nucleated ventricular cardiomyocytes constitute a transcriptionally homogenous cell population. Basic Res Cardiol [Internet]. 2019;114(5):1–13. Available from: doi: 10.1007/s00395-019-0744-z 31399804 PMC6689038

[60] Wang L, Yu P, Zhou B, Song J, Li Z, Zhang M, et al. Single-cell reconstruction of the adult human heart during heart failure and recovery reveals the cellular landscape underlying cardiac function. Nat Cell Biol [Internet]. 2020;22(January). Available from: doi: 10.1038/s41556-019-0446-7 31915373

[61] Cooley SM, Hamilton T, Deeds EJ, Ray JCJ. A novel metric reveals previously unrecognized distortion in dimensionality reduction of scRNA-Seq data. bioRxiv. 2019;1–36.

[62] Martinez O, Reyes-valdes MH. Defining diversity, specialization, and gene specificity in transcriptomes through information theory. Proc Natl Acad Sci. 2008;105(28):9709–14. doi: 10.1073/pnas.0803479105 18606989 PMC2443819

[63] Bagnoli JW, Ziegenhain C, Parekh S, Geuder J, Hellmann I, Janjic A, et al. Sensitive and powerful single-cell RNA sequencing using mcSCRB-seq. Nat Commun. 2018;9. doi: 10.1038/s41467-018-05347-6 30050112 PMC6062574

