# Supplementary figures and images for: Transcriptomic entropy benchmarks stem cell-derived cardiomyocyte maturation against endogenous tissue at single cell level
Source: PLoS Comput Biol. 2021 Sep 17;17(9):e1009305. doi: 10.1371/journal.pcbi.1009305 (PMC8448341; doi:10.1371/journal.pcbi.1009305)

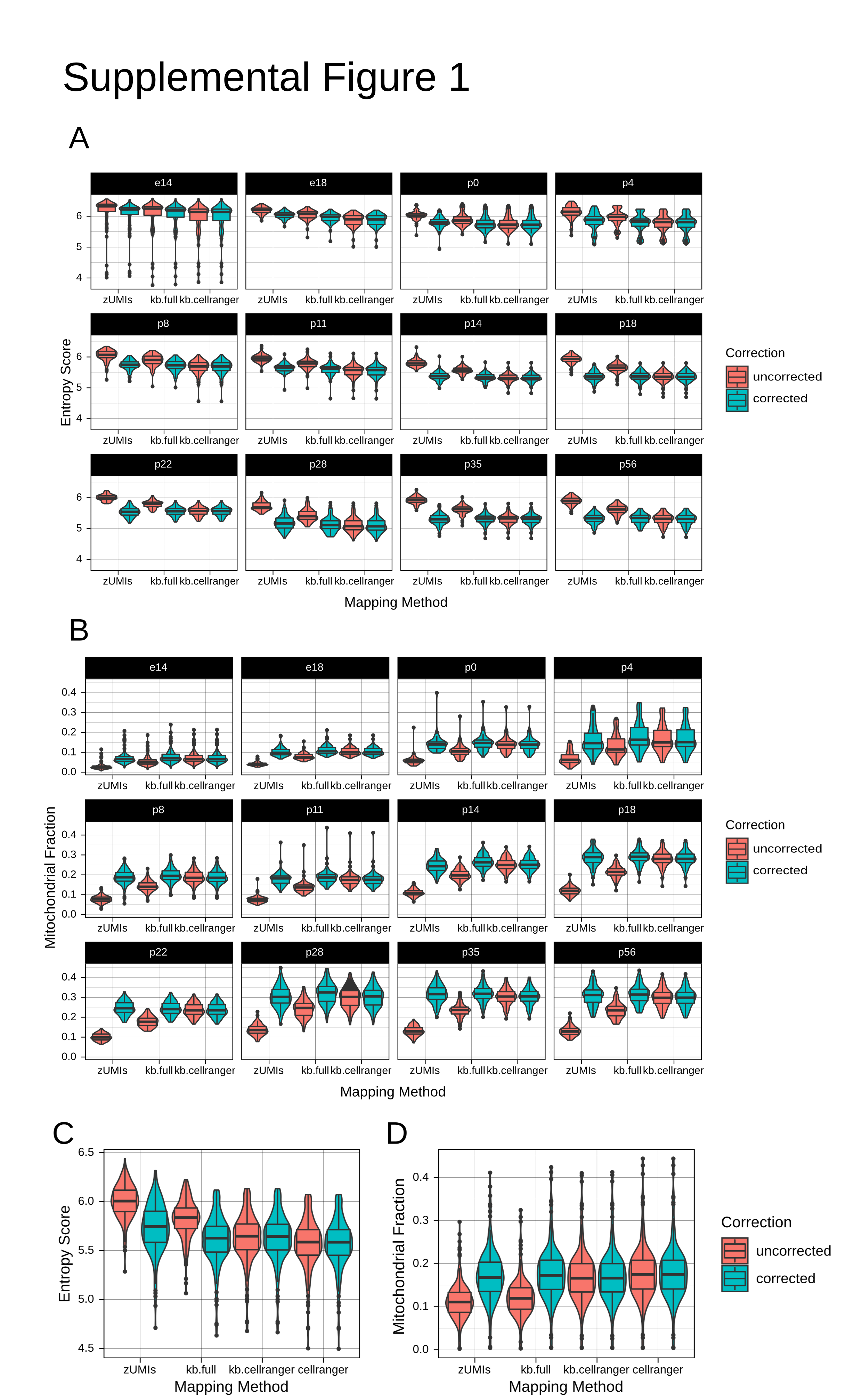

Supplement: S1 Fig — A. Entropy scores for the maturation reference dataset mapped by zUMIs, kallisto|bustools with the full reference, and kallisto|bustools with the CellRanger reference. Pre- and post-correction scores are shown. B. As in A, showing mitochondrial proportions. C. Entropy scores for the 10x Chromium heart dataset mapped by zUMIs, kallisto|bustools with the full reference, kallisto|bustools with the CellRanger reference, and CellRanger. D. As in C, showing mitochondrial proportions. (TIFF) [file pcbi.1009305.s001.tiff]

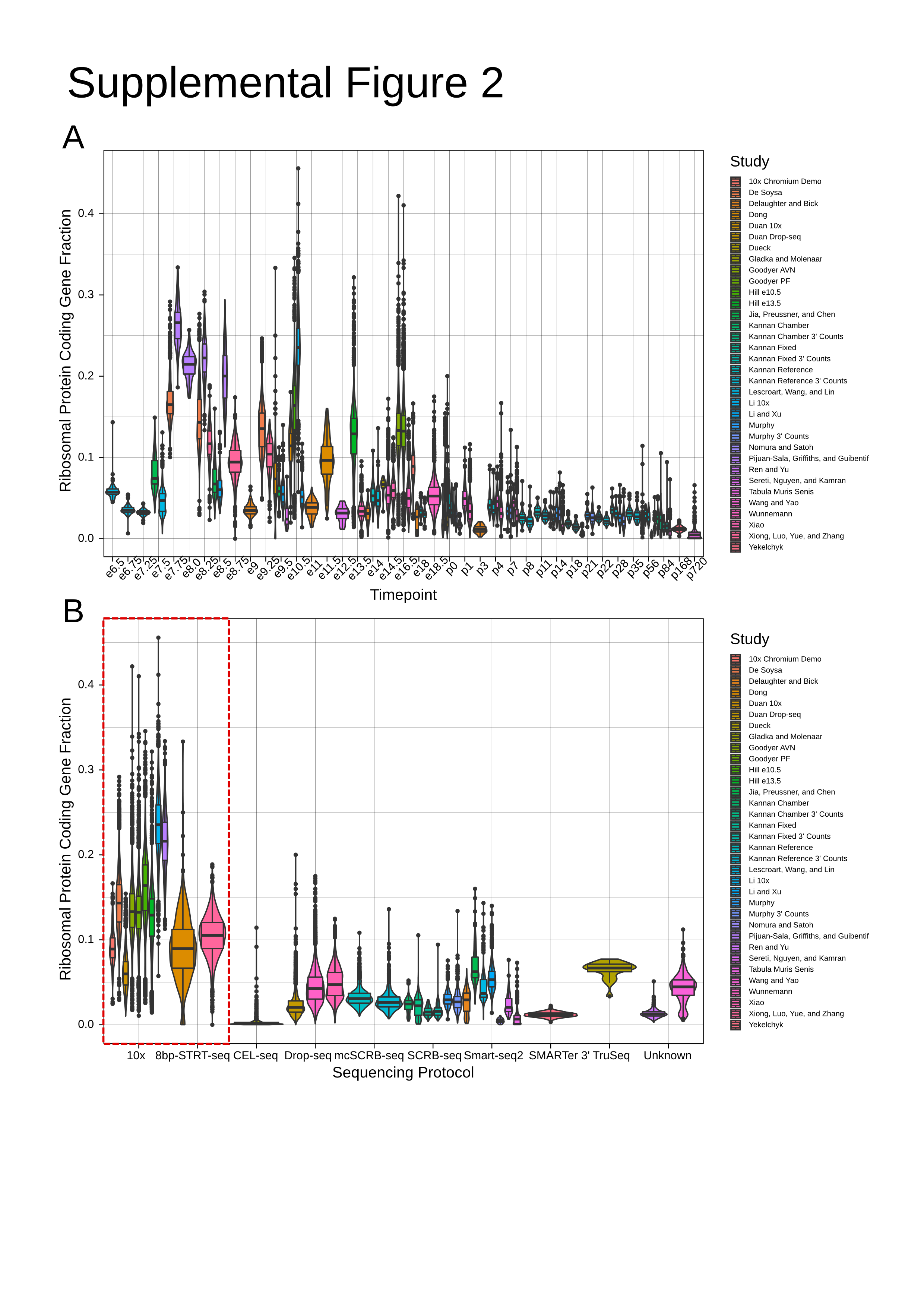

Supplement: S2 Fig — A. Proportion of ribosomal protein coding genes in mouse in vivo datasets, grouped by timepoint. B. Proportion of ribosomal protein coding genes in mouse in vivo datasets, grouped by library preparation method. 10x v1-v3 protocols have been coalesced together for the purposes of this figure. (TIFF) [file pcbi.1009305.s002.tiff]

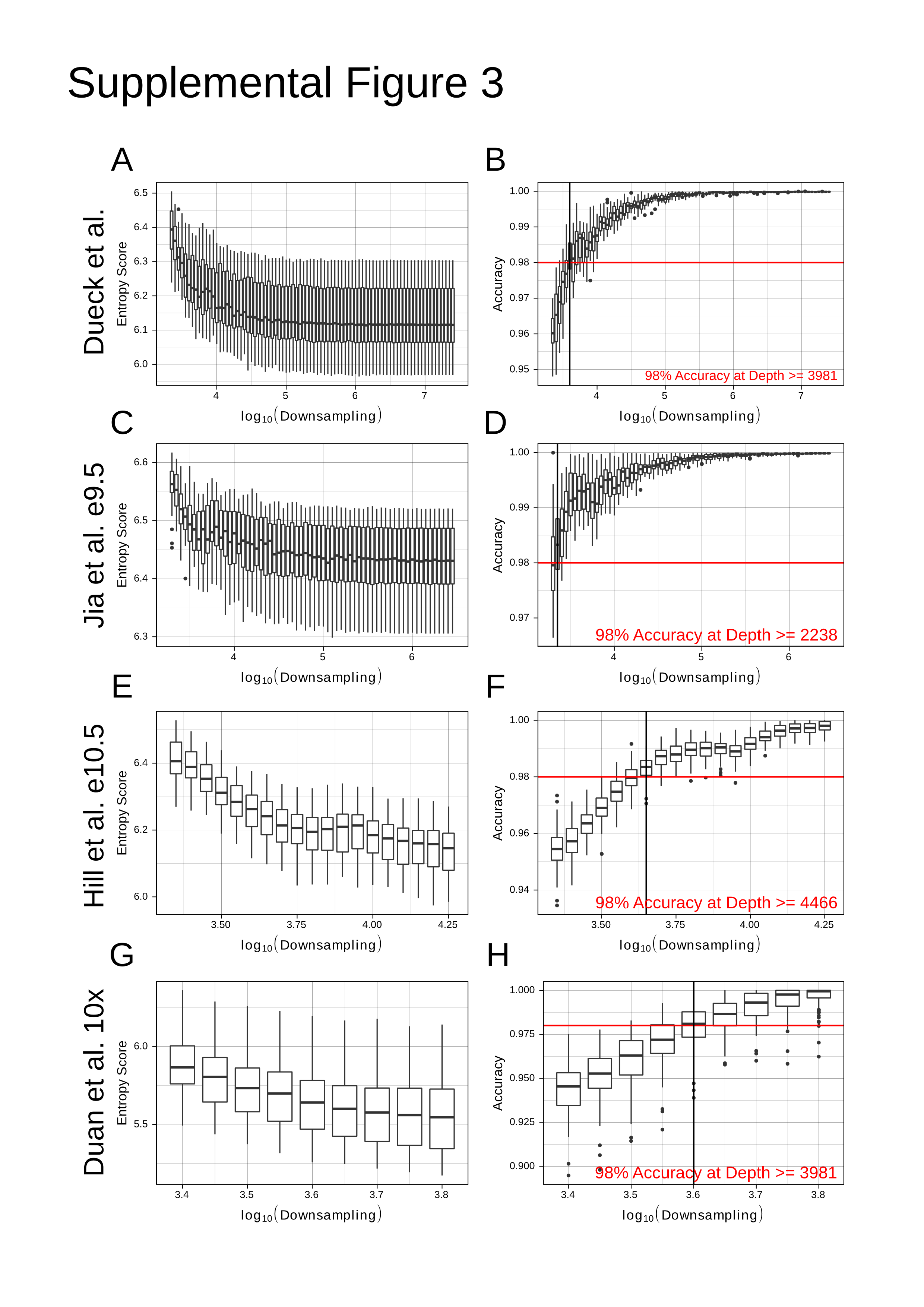

Supplement: S3 Fig — For each of four datasets, we performed subsampling and computed the entropy score as well as accuracy (calculated as deviation from baseline entropy score). At each stage, we included only cells with genes > 1000, and subsampled only to a depth where the median number of genes remained > 1000. Data is shown for A-B. Dueck et al. C-D. Jia et al. at e9.5. E-F. First 100 cells from Hill et al. at e10.5. G-H. First 100 cells from Duan et al. (TIFF) [file pcbi.1009305.s003.tiff]

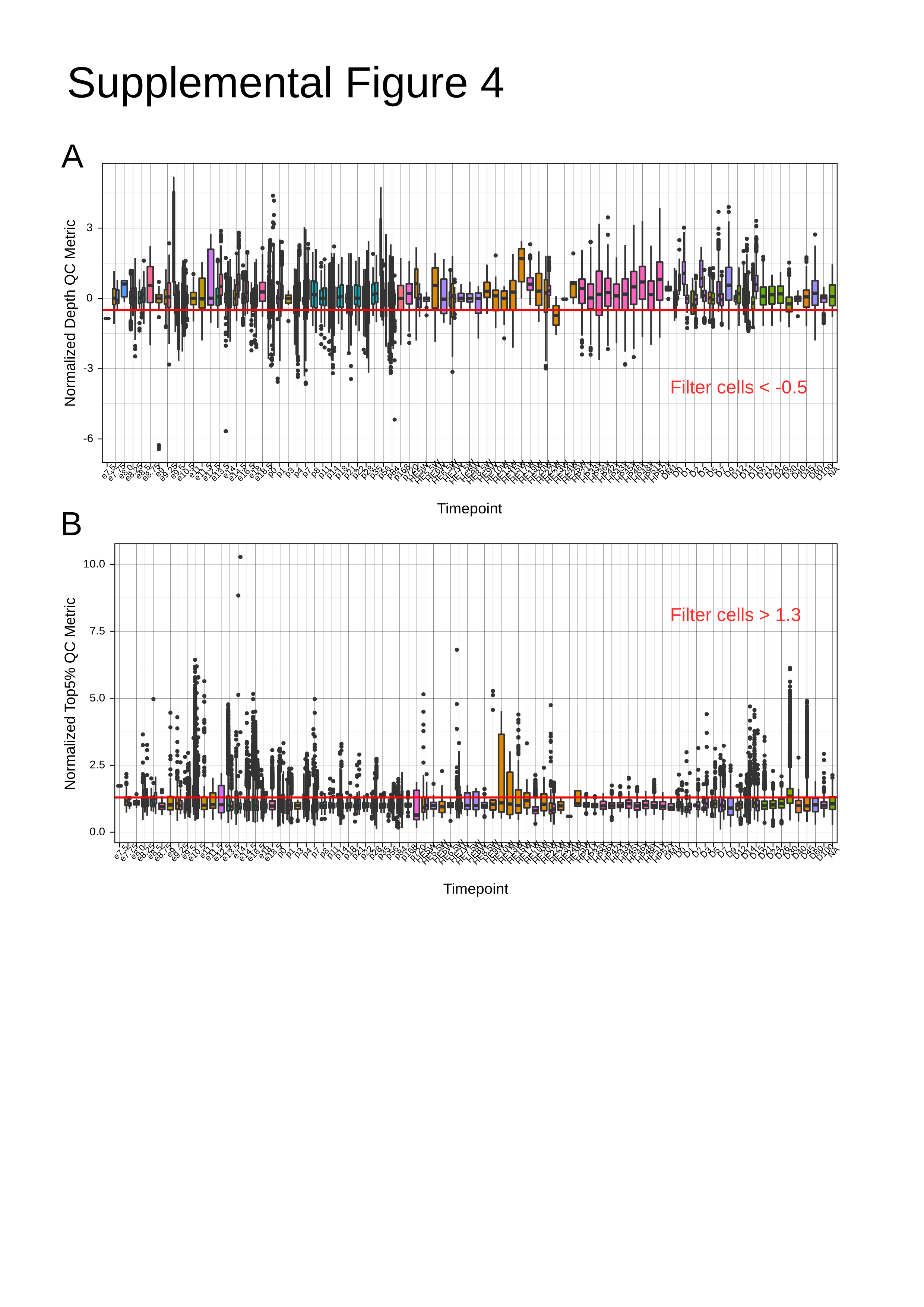

Supplement: S4 Fig — A. Normalized depth QC metric for all datasets. Red line indicates the threshold of −0.5. B. Normalized top 5 gene percentage metric for all datasets. Red line indicates the threshold of 1.3. (TIFF) [file pcbi.1009305.s004.tiff]

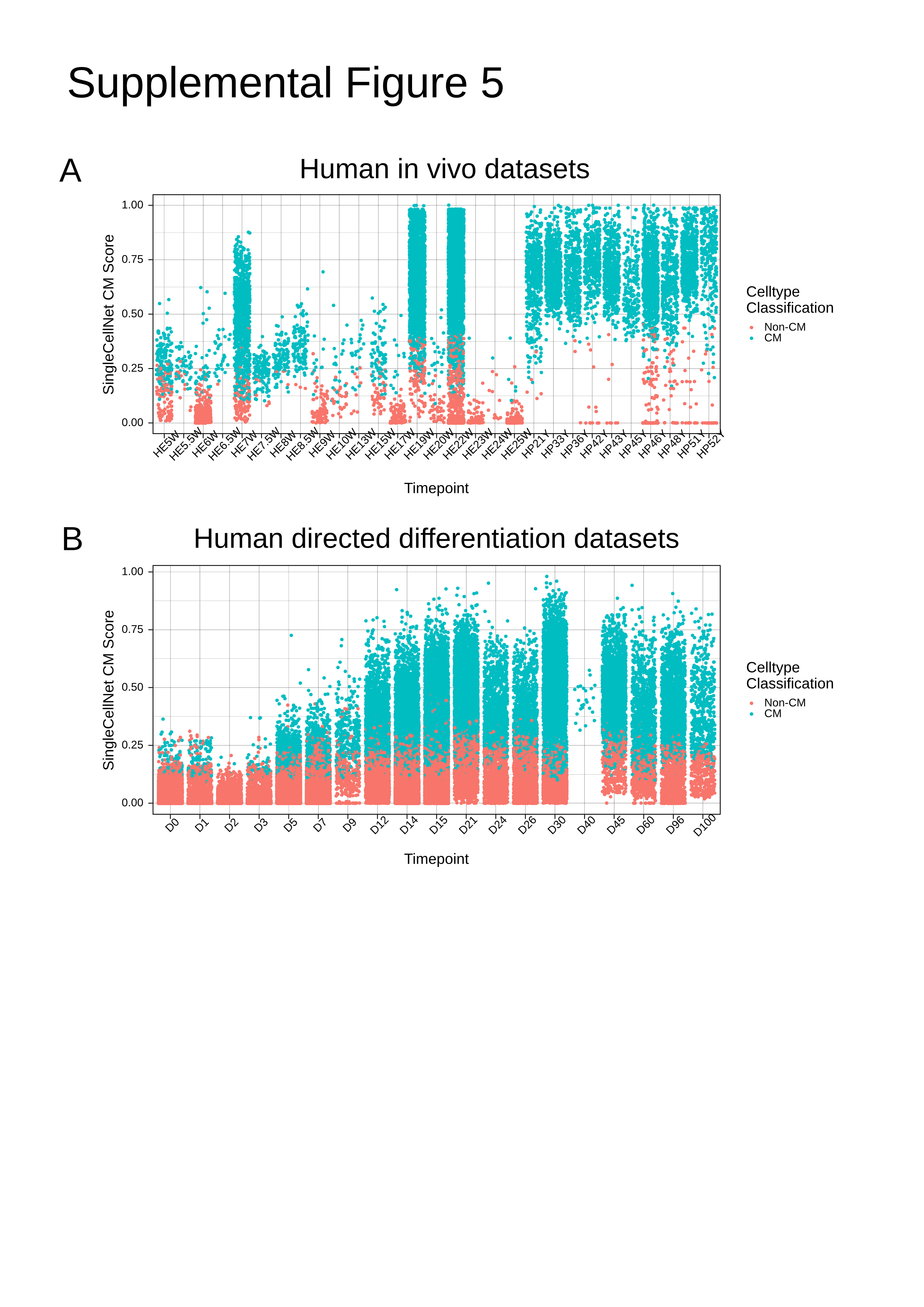

Supplement: S5 Fig — Cells are labeled based on whether their highest classification was for “cardiac muscle” or another celltype. A. For human in vivo datasets. B. For human in vitro directed differentiation datasets. (TIFF) [file pcbi.1009305.s005.tiff]

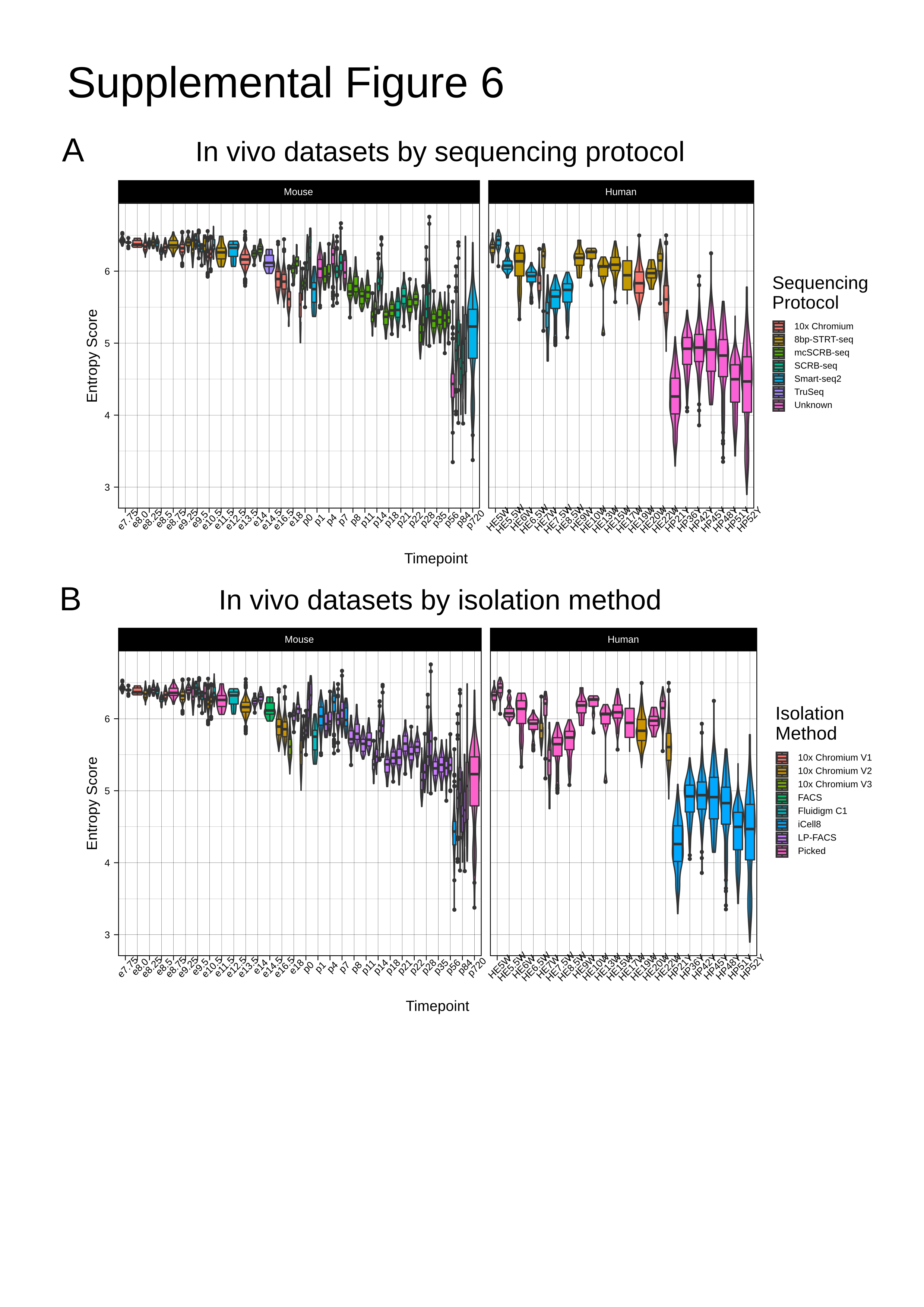

Supplement: S6 Fig — This figure corresponds to Fig 2B, but with boxplots coloured by A. sequencing protocol and B. isolation method. (TIFF) [file pcbi.1009305.s006.tiff]

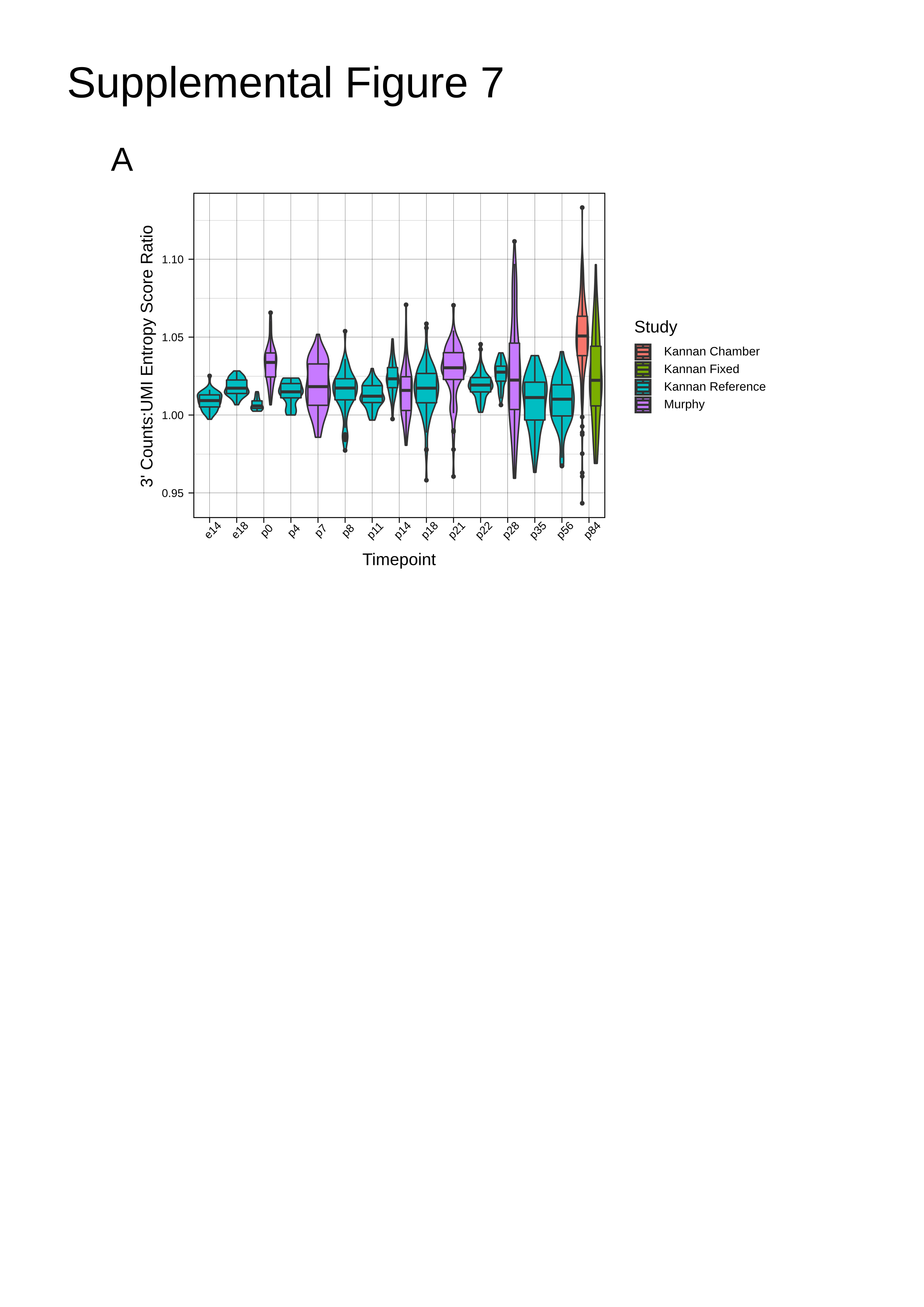

Supplement: S7 Fig — A. Ratio of entropy score for UMI datasets computed prior to vs. after UMI collapsing. (TIFF) [file pcbi.1009305.s007.tiff]

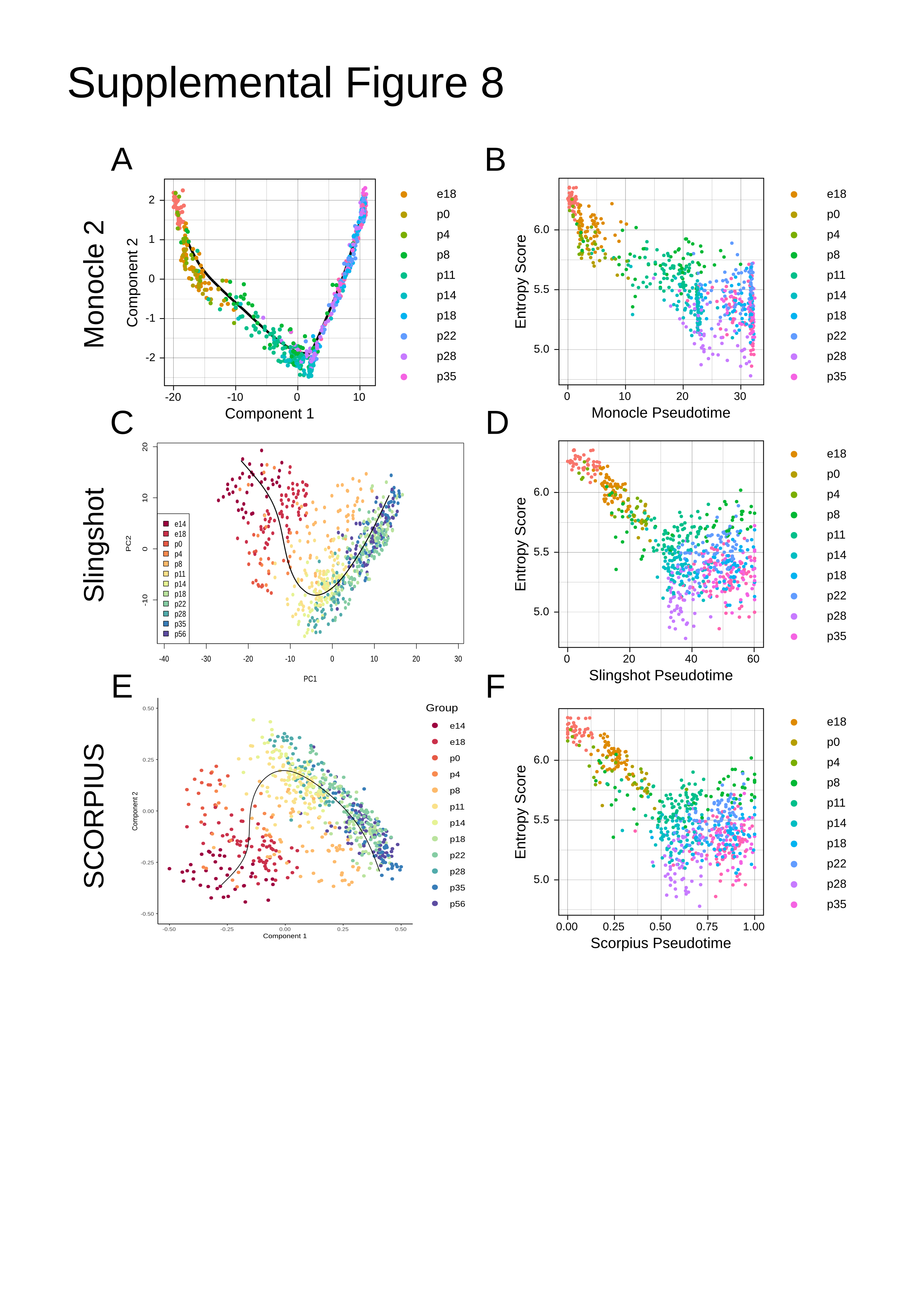

Supplement: S8 Fig — We reconstructed trajectories of our maturation reference dataset using A-B. Monocle 2, C-D. Slingshot, and E-F. SCORPIUS. (TIFF) [file pcbi.1009305.s008.tiff]

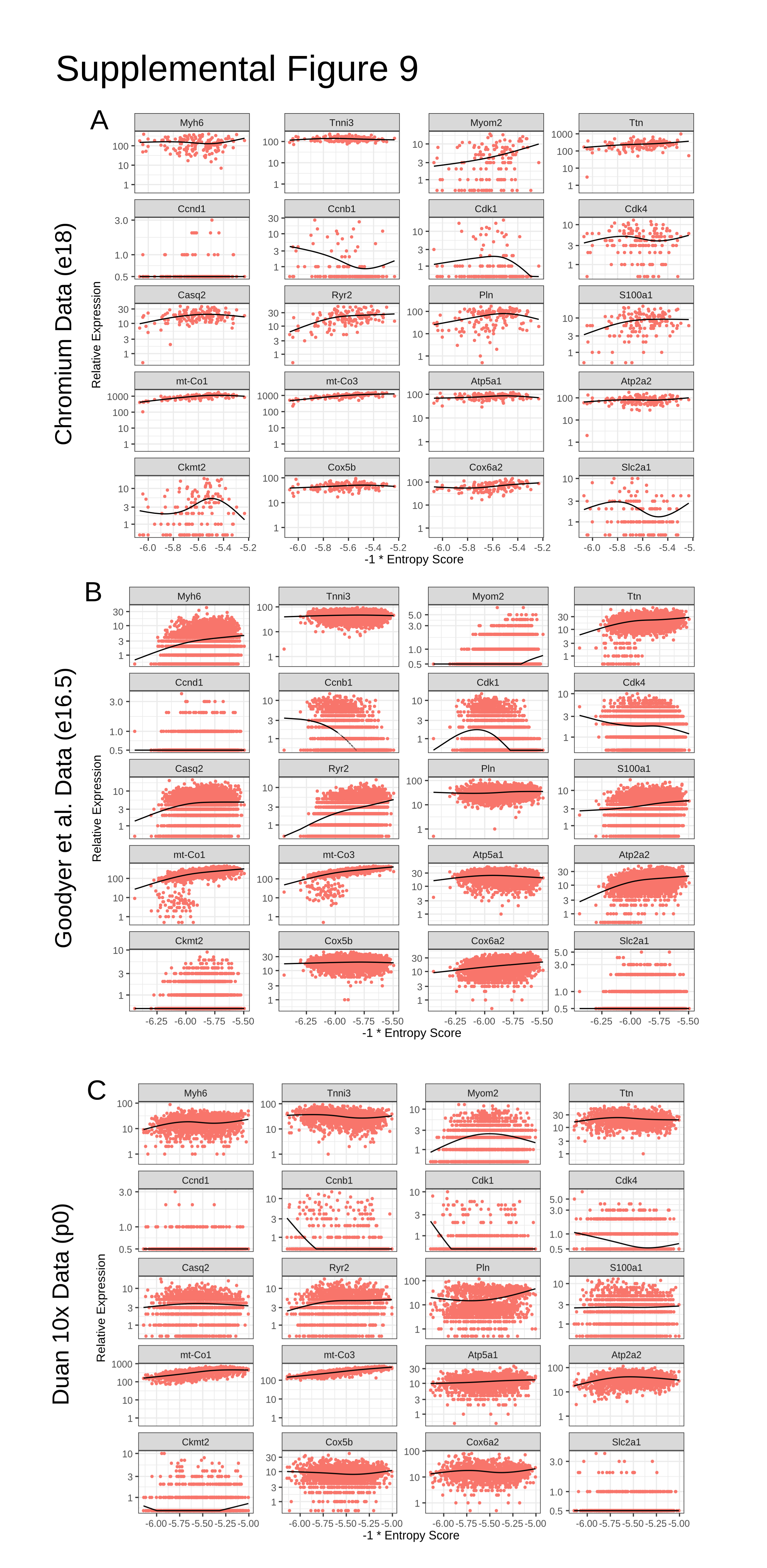

Supplement: S9 Fig — Gene trends across entropy score, as in Fig 3C, are plotted for A. 10x Chromium heart dataset, B. Goodyer et al., and C. Duan et al. (TIFF) [file pcbi.1009305.s009.tiff]

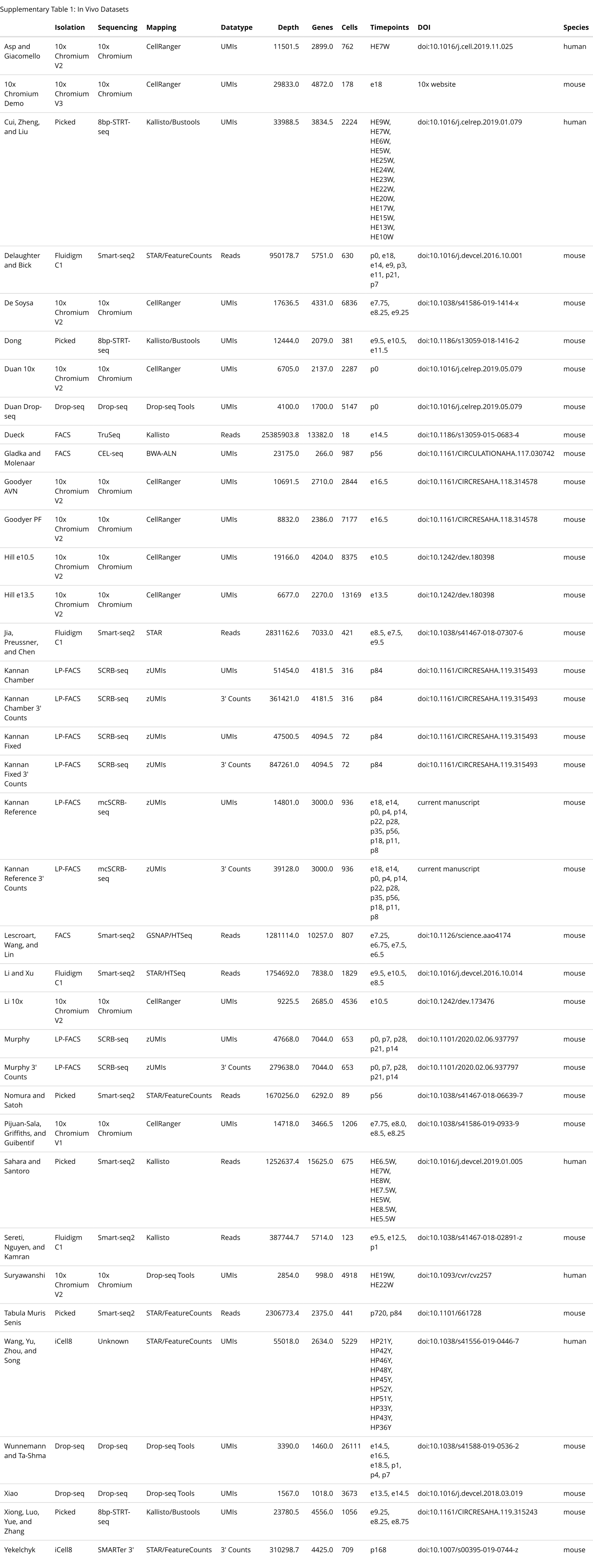

Supplement: S1 Table — (TIFF) [file pcbi.1009305.s010.tiff]

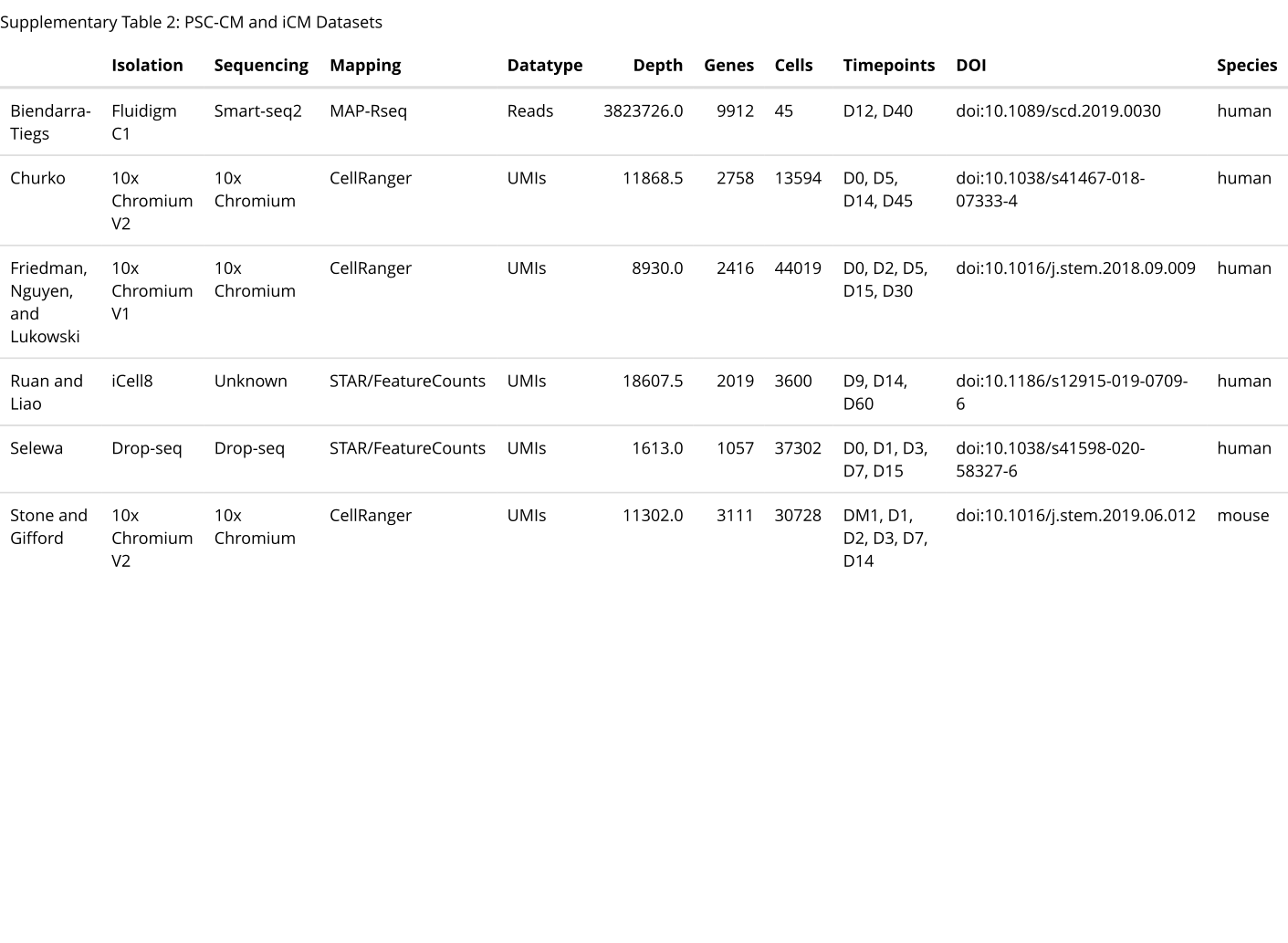

Supplement: S2 Table — (TIFF) [file pcbi.1009305.s011.tiff]
