# Supplementary material for: Transcriptomic entropy benchmarks stem cell-derived cardiomyocyte maturation against endogenous tissue at single cell level
Source: PLoS Comput Biol. 2021 Sep 17;17(9):e1009305. doi: 10.1371/journal.pcbi.1009305 (PMC8448341; doi:10.1371/journal.pcbi.1009305)
Supplement: S1 Text — (DOCX) [file pcbi.1009305.s012.docx]

**SUPPORTING TEXT 1**

**APPENDIX**

This appendix contains information about all of the datasets included for analysis in the study, including how they were acquired and processed. Metrics for each dataset are provided in **S1 and S2 Tables.** We have relevant data on Github at https://github.com/skannan4/cm-entropy-score. This includes an R workspace containing counts table for every dataset in this study, an R workspace containing just the final processed calculations (for lower memory usage), code for functions relevant to usage of entropy score, and code to reproduce the figures in the manuscript. If any additional information is required, we encourage direct inquiries and aim to respond as soon as possible.

***Mouse In Vivo***

*10x Chromium
10k Heart Cells from an E18 mouse (v3 chemistry)*

We downloaded the filtered feature/cell matrix from the 10x chromium datasets website (<https://support.10xgenomics.com/single-cell-gene-expression/datasets/3.0.0/heart_10k_v3>?). We subsequently performed UMAP + clustering using Seurat and selected the healthy cardiomyocytes as the cluster clearly expressing cardiomyocyte markers and having comparable/higher read/gene counts to other clusters.

*T. Yvanka de Soysa et al. (Casey Gifford, Deepak Srivastava)* (1)

*Single-cell analysis of cardiogenesis reveals basis for organ-level developmental defects*

We pulled the UMI data from the tables uploaded by the authors at GEO (GSE126128), selecting only wild-type cells at all available timepoints. We used the annotations provided by the authors as the source data to Extended Data Figure 1 to identify tissue, and selected out cells labeled as “myocardium.” We used the annotations provided by the authors as source data to Extended Figure 1 to annotate myocardial region, though we did not filter based on these annotations.

*Daniel DeLaughter and Alexander Bick et al. (Jonathan Seidman, Christine Seidman)* (2)

*Single-Cell Resolution of Temporal Gene Expression during Heart Development*

We pulled the FastQ data from the authors’ private database (<https://b2b.hci.utah.edu/gnomex/>) and mapped using STAR/FeatureCounts. We then performed T-SNE + clustering through Seurat and selected healthy cardiomyocytes as the clusters clearly expressing cardiomyocyte markers. However, we found that this data consistently had higher mitochondrial percentage at almost every timepoint compared to other datasets (most notably in the perinatal and postnatal timepoints). Thus, we discarded this dataset.

*Ji Dong et al. (Fuchou Tang)* (3)

*Single-cell RNA-seq analysis unveils a prevalent epithelial/mesenchymal hybrid state during mouse organogenesis*

The uploaded data of the authors does not contain mitochondrial reads. Therefore, we pulled and mapped the FastQ data from the four heart samples from ENA (PRJNA343327) using the FASTerQ approach with Kallisto/Bustools. Read 1 was used as the cdna read (base pairs 45 to 140, to avoid potential adaptor and poly A tails), while Read 2 was used for barcode (first 8 bp) and UMI (second 8 bp). Because of the short UMI length, we found that Kallisto/Bustools discarded many UMIs during counting. We thus used a custom script to count UMIs from the Kallisto/Bustools output.

*Jialei Duan et al. (Nikhil Munshi, Gary Hon)* (4)

*Rational Reprogramming of Cellular States by Combinatorial Perturbation*

The UMI data was pulled from the tables uploaded by the authors at GEO (GSE117795). We collated all of the in vivo samples, with the 10x and Drop-seq data being handled separately. We then performed UMAP + clustering through Seurat and selected healthy cardiomyocytes as the clusters clearly expressing cardiomyocyte markers while having comparable read counts to other clusters and comparable mitochondrial percentage (discarding clusters with notably high mitochondrial percentage or low genes). We could readily distinguish atrial and ventricular myocytes using myosin light chain isoforms, and annotated the cells accordingly. We found that the Drop-seq data had unusually high entropy, something we observed across multiple Drop-seq datasets (and perhaps owing to low depth); thus, we discarded the Drop-seq data.

*Hannah Dueck et al. (Junhyong Kim)* (5)

*Deep sequencing reveals cell-type-specific patterns of single-cell transcriptome variation*

The uploaded data of the authors does not contain mitochondrial reads. Therefore, we pulled the FastQ data from the cardiomyocyte samples from ENA (PRJNA244374) and then mapped with Kallisto (pseudo in batch mode with the –quant flag).

*Monika Gladka and Bas Molenaar et al. (Eva von Rooij)* (6)

*Single-Cell Sequencing of the Healthy and Diseased Heart Reveals Cytoskeleton-Associated Protein 4 as a New Modulator of Fibroblasts Activation*

Counts tables were kindly provided by the authors. We used UMAP + clustering through Seurat to select cardiomyocytes based on clusters expressing cardiomyocyte markers. We observed, however, that the dataset had very high mitochondrial percentages, often close to 90%. We thus discarded this dataset.

*William Goodyer (Sean Wu)* (7)

*Transcriptomic Profiling of the Developing Cardiac Conduction System at Single-Cell Resolution*

We pulled the UMI data from tables uploaded by the authors at GEO (GSE132658). The authors kindly provided us with the clustering used in the manuscript, which we used to select out cardiomyocytes. We used the PF (left and right) and AVN datasets as the metadata was not available for the SAN data.

*Matthew Hill et al. (James Martin)* (8)

*A cellular atlas of Pitx2-dependent cardiac development*

The UMI data was pulled from the tables uploaded by the authors (GSE131181), and the metadata tables were pulled from the same source. We used the authors’ generated clusters and selected clusters with high expression of cardiomyocyte markers. We subsequently selected only control cells from both timepoints.

*Guanshuai Jia, Jens Preussner, and Xi Chen et al. (Thomas Braun)* (9)

*Single cell RNA-seq and ATAC-seq analysis of cardiac progenitor cell transition states and lineage settlement*

The counts data was pulled from the authors’ Github (<https://github.com/loosolab/cardiac-progenitors>).

We used the data from both Isl and Nkx GFP lines.

*Suraj Kannan et al. (Chulan Kwon)* (10)

*Large Particle Fluorescence-Activated Cell Sorting Enables High-Quality Single-Cell RNA Sequencing and Functional Analysis of Adult Cardiomyocytes*

This data was generated at our lab and is available at GEO (GSE133640). We used both the multi-chamber study and the lived/fixed study and included all cells from both studies. Both 3’ counts and UMIs (output from zUMIs, using intronic and exonic reads) were used for analysis.

*Suraj Kannan et al. (Chulan Kwon)*

*Transcriptomic entropy enables quantification of cardiomyocyte maturation at single cell level*

This data (described in this manuscript) was generated at our lab and is available at GEO (GSE147807). Both 3’ counts and UMIs (output from zUMIs, using intronic and exonic reads) were used for anaysis.

*Fabienne Lescroart, Xiaonan Wang, and Xionghui Lin et al. (Cedric Blanpain)* (11)

*Defining the earliest step of cardiovascular lineage segregation by single-cell RNA-seq*

We download the counts data from the author’s private website (<http://singlecell.stemcells.cam.ac.uk/mesp1#data>). We subsequently selected only wild-type cells.

*Guang Li and Adele Xu et al. (Sean Wu)* (12)

*Transcriptomic Profiling Maps Anatomically Patterned Subpopulations among Single Embryonic Cardiac Cells*

We pulled the counts data uploaded by the authors at GEO (GSE76118). We subsequently performed TSNE + clustering through Seurat and selected clusters clearly identifiable as cardiomyocytes by marker gene expression. We selected all wild-type cells, including from the Nkx experiment.

*Guang Li et al. (Sean Wu)* (13)

*Single cell expression analysis reveals anatomical and cell cycle-dependent transcriptional shifts during heart development*

We pulled the UMI data uploaded by the authors at GEO (GSE122403). We subsequently performed UMAP + clustering through Seurat and selected clusters clearly identifiable as cardiomyocytes by marker gene expression.

*Sean Murphy et al. (Chulan Kwon)* (14)

*Single-Cell Analysis Identifies PGC1 as a Master Regulator of Cardiomyocyte Maturation*

This data was generated in our lab and is publicly available at GEO (GSE165917). We selected only the wild-type cardiomyocytes for further analysis. Both 3’ counts and UMIs (output from zUMIs, using intronic and exonic reads) were used for analysis.

*Seitaro Nomura and Masahiro Satoh et al. (Hiroyuki Aburatani, Issei Komuro)* (15)

*Cardiomyocyte gene programs encoding morphological and functional signatures in cardiac hypertrophy and failure*

The uploaded data of the authors does not contain mitochondrial reads. Therefore, we pulled and mapped the FastQ data from the sham cardiomyocytes from ENA (PRJNA376183) using STAR/FeatureCounts. However, we found that the dataset had a high percentage of mitochondrial reads; thus, we discarded this dataset.

*Blanca Pijuan-Sala, Jonathan Griffiths, and Caroline Guibentif et al. (John Marioni and Berthold Gottgens)* (16)

*A single-cell molecular map of mouse gastrulation and early organogenesis*

We pulled the UMI data following the instructions from the authors’ Github page (<https://github.com/MarioniLab/EmbryoTimecourse2018/blob/master/download/download.sh>). We subsequently used the authors’ labelings to select cells classified as “Cardiomyocyte.”

*Zongna Ren and Peng Yu et al. (Li Wang)* (17)

*Single-Cell Reconstruction of Progression Trajectory Reveals Intervention Principles in Pathological Cardiac Hypertrophy*

We pulled the UMI data uploaded by the authors at GEO (GSE120064). We subsequently performed TSNE + clustering through Seurat and selected clusters clearly identifiable as cardiomyocytes by marker gene expression. Many clusters had an abnormally high percentage of mitochondrial reads (e.g. >65%), so we selected only clusters with consistently reasonable mitochondrial percentages. We subsequently included only the control (non-disease) cells in our final analysis.

*Konstantina-Ioanna Sereti, Ngoc Nguyen, and Paniz Kamran et al. (Reza Ardehali)* (18)

*Analysis of cardiomyocyte clonal expansion during mouse heart development and injury*

The uploaded data of the authors does not contain mitochondrial reads. Therefore, we pulled the FastQ data from ENA (PRJNA427266) and remapped using Kallisto (pseudo in batch mode with the –quant flag). We found the p1 timepoint to be bimodal in terms of mitochondrial gene expression. However, because of the relatively small cell number and large fraction of poor quality cells, we found that our top5 filter did not catch all low quality cells. We thus also excluded all p1 cells with >30% mitochondrial reads.

*Tabula Muris Consortium* (19)

*A Single Cell Transcriptomic Atlas Characterizes Aging Tissues in the Mouse*

We pulled the BAM files for the Fluidigm studies from the publicly available AWS bucket (<https://registry.opendata.aws/tabula-muris-senis/>) and the annotations from the authors’ Figshare (<https://figshare.com/projects/Tabula_Muris_Senis/64982>). Specifically, for the latter, we used the metadata stored in the scanpy object, and matched the names of cells between this annotation and the raw data available through AWS. We focused only on the Fluidigm data for our study. We subsequently selected all cells from the “heart” tissue category. As we were unsure of the settings used to count with HTSeq, we recounted from the BAM files using FeatureCounts.

*Yin Wang and Fang Yao et al. (Li Wang)* (20)

*Single-cell analysis of murine fibroblasts identifies neonatal to adult switching that regulates cardiomyocyte maturation*

We pulled the UMI uploaded by the authors at GEO (GSE122706). We subsequently performed TSNE+clustering through Seurat and selected clusters clearly identifiable as cardiomyocytes. However, we observed that the p14 cells demonstrates particularly high mitochondrial percentages, likely due to use of non-perfusion-based dissociation at this relatively late stage. We therefore discarded this timepoint.

*Florian Wunnemann (Gregor Andelfinger)*

Counts table and metadata were kindly provided by the authors. We used UMAP + clustering through Seurat to select cardiomyocytes based on clusters expressing cardiomyocyte markers and having comparable read and gene counts to other clusters. We additionally annotated clusters as atrial or ventricular, though we analyzed both sets of cells. However, as with several other Drop-seq datasets, we found that the data had unusually high entropy (and perhaps owing to low depth); thus, we discarded this dataset.

*Yang Xiao et al. (James Martin)* (21)

*Hippo Signaling Plays an Essential Role in Cell State Transitions during Cardiac Fibroblast Development*

We pulled the UMI data uploaded by the authors are GEO (GSE100861). The metadata was kindly provided by the authors, and included the clustering used in the manuscript. We selected wild-type cells in the cardiomyocyte cluster. However, as with several other Drop-seq datasets, we found that the data had unusually high entropy (and perhaps owing to low depth); thus, we discarded this dataset.

*Haiqing Xiong, Yingjie Luo, Yanzhu Yue, and Jiejie Zhang et al. (Albin He)* (22)

*Single-Cell Transcriptomics Reveals Chemotaxis-Mediated Intraorgan Crosstalk During Cardiogenesis*

The uploaded data of the authors does not contain mitochondrial reads. Therefore, we pulled and mapped the FastQ data from the four heart samples from ENA (PRJNA429249) using the FASTerQ approach with Kallisto/Bustools. Read 1 was used as the cdna read (base pairs 45 to 140, to avoid potential adaptor and poly A tails), while Read 2 was used for barcode (first 8 bp) and UMI (second 8 bp). Because of the short UMI length, we found that Kallisto/Bustools discarded many UMIs during counting. We thus used a custom script to count UMIs from the Kallisto/Bustools output.

*Michail Yekelchyk et al. (Thomas Braun)* (23)

*Mono- and multi-nucleated ventricular cardiomyocytes constitute a transcriptionally homogenous cell population*

We pulled the mapped BAM files of wild-type cells from ENA (PRJEB29049) and recounted using FeatureCounts. However, we found that the dataset had high mitochondrial read percentage, and thus we discarded this dataset.

***Human In Vivo***

*Michaela Asp and Stefania Giacomello et al. (Joakim Lundberg)* (24)

*A Spatiotemporal Organ-Wide Gene Expression and Cell Atlas of the Developing Human Heart*

We pulled the UMI data and annotations for the single cell sequencing data from the authors’ website (<https://www.spatialresearch.org/resources-published-datasets/doi-10-1016-j-cell-2019-11-025/>). We used the authors’ clustering and selected all cells classified as cardiomyocytes.

*Yueli Cui, Yuxuan Zheng, and Xixi Liu et al. (Jie Qiao, Fuchou Tang)* (25)

*Single-Cell Transcriptome Analysis Maps the Developmental Track of the Human Heart*

The uploaded data of the authors does not contain mitochondrial reads. Therefore, we pulled and mapped the FastQ data from the four heart samples from ENA (PRJNA415637) using the FASTerQ approach with Kallisto/Bustools. Read 1 was used as the cdna read (base pairs 45 to 140, to avoid potential adaptor and poly A tails), while Read 2 was used for barcode (first 8 bp) and UMI (second 8 bp). We then performed UMAP + clustering in Seurat, and selected clusters that clearly expressed cardiomyocyte markers. Because of the short UMI length, we found that Kallisto/Bustools discarded many UMIs during counting. We thus used a custom script to count UMIs from the Kallisto/Bustools output. We subsequently found that some, though not all, samples had unusually high mitochondrial percentages (namely – HE13W RV; HE17W AV, LA, LV, TV; HE20W RA; HE25W all samples). We also removed samples as post-filtering, there were too few for useful analysis (namely – HE23W, HE24W).

*Makoto Sahara and Federica Santoro et al. (Kenneth Chien)* (26)

*Population and Single-Cell Analysis of Human Cardiogenesis Reveals Unique LGR5 Ventricular Progenitors in Embryonic Outflow Tract*

We downloaded the raw FastQ data from ENA (PRJNA510181), and subsequently mapped using Kallisto (pseudo in batch mode with the –quant flag). We had some concerns about several timepoints in this study due to high mitochondrial percentage. We eliminated some (namely – HE7W OFT, A; and HE8W); however, we are somewhat unsure about the quality of the data HE7W onwards.

*Hemant Suryawanshi et al. (Jill Buyon, Thomas Tuschl)* (27)

*Cell atlas of the foetal human heart and implications for autoimmune-mediated congenital heart block*

The UMI data for the wild-type hearts was kindly provided by the authors as a Seurat object, and also included the authors’ UMAP clustering. We utilized their clustering to identify and select cardiomyocytes; we additionally filtered out cells with notably high mitochondrial percentage or low counts/genes.

*Li Wang, Peng Yu, Bingying Zhou, and Jiangping Song et al. (Shengshou Hu)* (28)

*Single-cell reconstruction of the adult human heart during heart failure and recovery reveals the cellular landscape underlying cardiac function*

We download the UMI data and phenotype tables provided by the authors at GEO (GSE109816), selecting only the healthy heart tissue data. We used the authors’ provided metadata to select cardiomyocytes. We found that three of the four ventricular donors had extremely high mitochondrial percentages (~70%). Very little is currently known about human adult CMs, so it is difficult to assess the validity of this range. However, one ventricular sample had a lower percentage, which also matched the atrial samples. We chose to therefore exclude the three samples with extremely high mitochondrial percentage, pending discovery of further information.

***Human Directed Differentiation***

*Sherri Biendarra-Tiegs et al. (Timothy Nelson)* (29)

*Single-Cell RNA-Sequencing and Optical Electrophysiology of Human Induced Pluripotent Stem Cell-Derived Cardiomyocytes Reveal Discordance Between Cardiac Subtype-Associated Gene Expression Patterns and Electrophysiological Phenotypes*

The counts data was kindly provided by the authors. We selected cardiomyocytes using the annotations provided in Figure 4 of the manuscript.

*Jared Churko et al. (Nathan Salomonis, Joseph Wu)* (30)

*Defining human cardiac transcription factor hierarchies using integrated single-cell heterogeneity analysis*

We pulled the UMI data for all timepoints from the authors’ Synapse (<https://www.synapse.org/#!Synapse:syn18078447/files/>), using the V2 chemistry.

*Clayton Friedman, Quan Nguyen, and Samuel Lukowski et al. (Joseph Powell, Nathan Palpant)* (31)

*Single-Cell Transcriptomic Analysis of Cardiac Differentiation from Human PSCs Reveals HOPX-Dependent Cardiomyocyte Maturation*

We pulled the UMI data for all timepoints from the authors’ processed data upload at ArrayExpress (E-MTAB-6268, <https://www.ebi.ac.uk/arrayexpress/experiments/E-MTAB-6268/samples/>).

*Kathryn Gerbin, Tanya Grancharova, Rory Donovan-Maiye, and Melissa Hendershott et al. (Ruwanthi Gunawardane)* (32)

*Cell states beyond transcriptomics: integrating structural organization and gene expression in hiPSC-derived cardiomyocytes*

We pulled the UMI data for all timepoints from the authors from the authors’ data upload at Quilt (<https://open.quiltdata.com/b/allencell/tree/aics/integrated_transcriptomics_structural_organization_hipsc_cm/scrnaseq_data/>). However, we found that Batch 2 (composed of D0/D93/D96 cells) was under our approximate depth threshold (with a potentially similar issue to our Drop-seq data); thus, we focused on Batch 1. We performed TSNE + clustering through Seurat to identify cardiomyocytes.

*Elisa Giacomelli, Viviana Meraviglia, and Giulia Campostrini et al. (Valeria Orlova, Milena Bellin and Christine Mummery)* (33)

*Human-iPSC-Derived Cardiac Stromal Cells Enhance Maturation in 3D Cardiac Microtissues and Reveal Non-cardiomyocyte Contributions to Heart Disease*

We pulled the UMI data uploaded by the authors at GEO (GSE147694). We subsequently performed TSNE+clustering in Seurat to identify cardiomyocytes. We included only the control CMs (e.g. not co-cultured with ECs) in our final analysis.

*Hang Ruan and Yingnan Liao et al. (Leng Han, Li Wang)* (34)

*Single-cell reconstruction of differentiation trajectory reveals a critical role of ETS1 in human cardiac lineage commitment*

We pulled the UMI for the D9, D14, and D60 timepoints from the tables uploaded by the authors at GEO (GSE129987).

*Adam Selewa et al. (Sebastian Pott, Anindita Basu)* (35)

*Systematic Comparison of High-throughput Single-Cell and Single-Nucleus Transcriptomes during Cardiomyocyte Differentiation*

We pulled the UMI data for the Drop-seq samples from the tables uploaded by the authors at GEO (GSE129096). However, as with several other Drop-seq datasets, we found that the data had unusually high entropy (and perhaps owing to low depth); thus, we discarded this dataset.

*Ana Silva et al. (Todd McDevitt)* (36)

*Developmental co-emergence of cardiac and gut tissues modeled by human iPSC-derived organoids*

The UMI counts data was kindly provided by the authors. We performed TSNE + clustering through Seurat to identify cardiomyocytes.

***Mouse Direct Reprogramming***

*Nicole R. Stone and Casey A. Gifford et al. (Deepak Srivastava)* (37)

*Context-Specific Transcription Factor Functions Regulate Epigenomic and Transcriptional Dynamics during Cardiac Reprogramming*

We pulled the UMI data for the 3’ study from tables uploaded by the authors at GEO (GSE131328). We subsequently performed TSNE + clustering through Seurat and compared the generated clusters to those in the manuscript to assign cells into the putative trajectory reprogramming groups (as done by the authors).

**REFERENCES**

1. de Soysa TY, Ranade SS, Okawa S, Ravichandran S, Huang Y, Salunga HT, et al. Single-cell analysis of cardiogenesis reveals basis for organ-level developmental defects. Nature [Internet]. 2019;572(7767):120–4. Available from: http://dx.doi.org/10.1038/s41586-019-1414-x

2. DeLaughter DM, Bick AG, Wakimoto H, McKean D, Gorham JM, Kathiriya IS, et al. Single-Cell Resolution of Temporal Gene Expression during Heart Development. Dev Cell [Internet]. 2016;39(4):480–90. Available from: http://dx.doi.org/10.1016/j.devcel.2016.10.001

3. Dong J, Hu Y, Fan X, Wu X, Mao Y, Hu B, et al. Single-cell RNA-seq analysis unveils a prevalent epithelial/mesenchymal hybrid state during mouse organogenesis. Genome Biol. 2018;19(1):1–20.

4. Duan J, Li B, Bhakta M, Xie S, Zhou P, Munshi N V., et al. Rational Reprogramming of Cellular States by Combinatorial Perturbation. Cell Rep [Internet]. 2019;27(12):3486-3499.e6. Available from: https://doi.org/10.1016/j.celrep.2019.05.079

5. Dueck H, Khaladkar M, Kim TK, Spaethling JM, Francis C, Suresh S, et al. Deep sequencing reveals cell-type-specific patterns of single-cell transcriptome variation. Genome Biol [Internet]. 2015;16(1):1–17. Available from: http://dx.doi.org/10.1186/s13059-015-0683-4

6. Gladka MM, Molenaar B, de Ruiter H, van der Elst S, Tsui H, Versteeg D, et al. Single-Cell Sequencing of the Healthy and Diseased Heart Reveals Ckap4 as a New Modulator of Fibroblasts Activation. Circulation [Internet]. 2018;CIRCULATIONAHA.117.030742. Available from: http://circ.ahajournals.org/lookup/doi/10.1161/CIRCULATIONAHA.117.030742

7. Goodyer WR, Beyersdorf BM, Paik DT, Tian L, Li G, Buikema JW, et al. Transcriptomic profiling of the developing cardiac conduction system at single-cell resolution. Circ Res. 2019;125(4):379–97.

8. Hill MC, Kadow ZA, Li L, Tran TT, Wythe JD, Martin JF. A cellular atlas of Pitx2-dependent cardiac development. Dev. 2019;146(12):1–12.

9. Jia G, Preussner J, Chen X, Guenther S, Yuan X, Yekelchyk M, et al. Single cell RNA-seq and ATAC-seq analysis of cardiac progenitor cell transition states and lineage settlement. Nat Commun [Internet]. 2018;9(1). Available from: http://dx.doi.org/10.1038/s41467-018-07307-6

10. Kannan S, Miyamoto M, Lin BL, Zhu R, Murphy S, Kass D, et al. Large particle fluorescence-activated cell sorting enables high-quality single-cell RNA sequencing and functional analysis of adult cardiomyocytes. Circ Res. 2019;

11. Lescroart F, Wang X, Lin X, Swedlund B, Gargouri S, Sànchez-Dànes A, et al. Defining the earliest step of cardiovascular lineage segregation by single-cell RNA-seq. Science (80- ). 2018;359(6380):1177–81.

12. Li G, Xu A, Sim S, Priest JR, Tian X, Khan T, et al. Transcriptomic Profiling Maps Anatomically Patterned Subpopulations among Single Embryonic Cardiac Cells. Dev Cell [Internet]. 2016;39(4):491–507. Available from: http://dx.doi.org/10.1016/j.devcel.2016.10.014

13. Li G, Tian L, Goodyer W, Kort EJ, Buikema JW, Xu A, et al. Single cell expression analysis reveals anatomical and cell cycle-dependent transcriptional shifts during heart development. Dev. 2019;146(12).

14. Murphy SA, Miyamoto M, Kervadec A, Kannan S, Tampakakis E, Kambhampati S, et al. PGC1/PPAR drive cardiomyocyte maturation at single cell level via YAP1 and SF3B2. Nat Commun [Internet]. 2021;12(1):1–12. Available from: http://dx.doi.org/10.1038/s41467-021-21957-z

15. Nomura S, Satoh M, Fujita T, Higo T, Sumida T, Ko T, et al. Cardiomyocyte gene programs encoding morphological and functional signatures in cardiac hypertrophy and failure. Nat Commun [Internet]. 2018;1–17. Available from: http://dx.doi.org/10.1038/s41467-018-06639-7

16. Pijuan-Sala B, Griffiths JA, Guibentif C, Hiscock TW, Jawaid W, Calero-Nieto FJ, et al. A single-cell molecular map of mouse gastrulation and early organogenesis. Nature. 2019;566(7745):490–5.

17. Ren Z, Yu P, Li D, Li Z, Liao Y, Wang Y, et al. Single-Cell Reconstruction of Progression Trajectory Reveals Intervention Principles in Pathological Cardiac Hypertrophy. Circulation. 2020;1704–19.

18. Sereti KI, Nguyen NB, Kamran P, Zhao P, Ranjbarvaziri S, Park S, et al. Analysis of cardiomyocyte clonal expansion during mouse heart development and injury. Nat Commun [Internet]. 2018;9(1). Available from: http://dx.doi.org/10.1038/s41467-018-02891-z

19. Consortium TM. Single-cell transcriptomics of 20 mouse organs creates a Tabula Muris. Nature. 2018;562.

20. Wang Y, Yao F, Wang L, Li Z, Ren Z, Li D, et al. Single-cell analysis of murine fibroblasts identifies neonatal to adult switching that regulates cardiomyocyte maturation. Nat Commun [Internet]. 2020;11(1). Available from: http://dx.doi.org/10.1038/s41467-020-16204-w

21. Xiao Y, Hill MC, Zhang M, Martin TJ, Morikawa Y, Wang S, et al. Hippo Signaling Plays an Essential Role in Cell State Transitions during Cardiac Fibroblast Development. Dev Cell [Internet]. 2018;45(2):153-169.e6. Available from: http://dx.doi.org/10.1016/j.devcel.2018.03.019

22. Xiong H, Luo Y, Yue Y, Zhang J, Ai S, Li X, et al. Single-Cell Transcriptomics Reveals Chemotaxis-Mediated Intraorgan Crosstalk during Cardiogenesis. Circ Res. 2019;125(4):398–410.

23. Yekelchyk M, Guenther S, Preussner J, Braun T. Mono ‑ and multi ‑ nucleated ventricular cardiomyocytes constitute a transcriptionally homogenous cell population. Basic Res Cardiol [Internet]. 2019;114(5):1–13. Available from: https://doi.org/10.1007/s00395-019-0744-z

24. Asp M, Giacomello S, Larsson L, Wu C, Fürth D, Qian X, et al. A Spatiotemporal Organ-Wide Gene Expression and Cell Atlas of the Developing Human Heart. Cell. 2019;179(7):1647-1660.e19.

25. Cui Y, Zheng Y, Liu X, Yan L, Fan X, Yong J, et al. Single-Cell Transcriptome Analysis Maps the Developmental Track of the Human Heart. Cell Rep [Internet]. 2019;26(7):1934-1950.e5. Available from: https://doi.org/10.1016/j.celrep.2019.01.079

26. Sahara M, Santoro F, Sohlmér J, Zhou C, Witman N, Leung CY, et al. Population and Single-Cell Analysis of Human Cardiogenesis Reveals Unique LGR5 Ventricular Progenitors in Embryonic Outflow Tract. Dev Cell. 2019;48(4):475-490.e7.

27. Suryawanshi H, Clancy R, Morozov P, Halushka MK, Buyon JP, Tuschl T. Cell atlas of the foetal human heart and implications for autoimmune-mediated congenital heart block. Cardiovasc Res. 2020;116(8):1446–57.

28. Wang L, Yu P, Zhou B, Song J, Li Z, Zhang M, et al. Single-cell reconstruction of the adult human heart during heart failure and recovery reveals the cellular landscape underlying cardiac function. Nat Cell Biol [Internet]. 2020;22(January). Available from: http://dx.doi.org/10.1038/s41556-019-0446-7

29. Biendarra-Tiegs SM, Li X, Ye D, Brandt EB, Ackerman MJ, Nelson TJ. Single-Cell RNA-Sequencing and Optical Electrophysiology of Human Induced Pluripotent Stem Cell-Derived Cardiomyocytes Reveal Discordance between Cardiac Subtype-Associated Gene Expression Patterns and Electrophysiological Phenotypes. Stem Cells Dev. 2019;28(10):659–73.

30. Churko JM, Garg P, Treutlein B, Venkatasubramanian M, Wu H, Lee J, et al. Defining human cardiac transcription factor hierarchies using integrated single-cell heterogeneity analysis. Nat Commun. 2018;9(1).

31. Friedman CE, Nguyen Q, Lukowski SW, Helfer A, Chiu HS, Voges HK, et al. Analysis of cardiac differentiation at single cell resolution reveals a requirement of hypertrophic signaling for HOPX transcription. bioRxiv. 2017;

32. Gerbin K, Grancharova T, Donovan-Maiye R, Hendershott M, Brown J, Dinh S, et al. Cell states beyond transcriptomics: integrating structural organization and gene expression in hiPSC-derived cardiomyocytes. 2020;

33. Giacomelli E, Meraviglia V, Campostrini G, Cochrane A, Cao X, van Helden RWJ, et al. Human-iPSC-Derived Cardiac Stromal Cells Enhance Maturation in 3D Cardiac Microtissues and Reveal Non-cardiomyocyte Contributions to Heart Disease. Cell Stem Cell. 2020;26(6):862-879.e11.

34. Ruan H, Liao Y, Ren Z, Mao L, Yao F, Yu P, et al. Single-cell reconstruction of differentiation trajectory reveals a critical role of ETS1 in human cardiac lineage commitment. BMC Biol. 2019;17(1):1–16.

35. Selewa A, Dohn R, Eckart H, Lozano S, Xie B, Gauchat E, et al. Systematic Comparison of High- throughput Single-Cell and Single- Nucleus Transcriptomes during Cardiomyocyte Differentiation. Sci Rep. 2020;10:1–13.

36. Silva AC, Matthys OB, Joy DA, Kauss MA, Natarajan V, Lai MH, et al. Developmental co-emergence of cardiac and gut tissues modeled by human iPSC- 2 derived organoids. bioRxiv. 2021;

37. Stone NR, Gifford CA, Thomas R, Ivey KN, Pollard KS, Stone NR, et al. Context-Specific Transcription Factor Functions Regulate Epigenomic and Transcriptional Dynamics during Cardiac Reprogramming. Cell Stem Cell [Internet]. 2019;25(1):87-102.e9. Available from: https://doi.org/10.1016/j.stem.2019.06.012
