# Supplementary material for: Transcriptomic entropy benchmarks stem cell-derived cardiomyocyte maturation against endogenous tissue at single cell level
Source: PLoS Comput Biol. 2021 Sep 17;17(9):e1009305. doi: 10.1371/journal.pcbi.1009305 (PMC8448341; doi:10.1371/journal.pcbi.1009305)
Supplement: Attachment — Submitted filename: 112939_1_rebuttal_2012791_qppbk4.pdf [file pcbi.1009305.s013.pdf]

Supplementary Information for

Transcriptomic entropy benchmarks stem cell-derived cardiomyocyte maturation against endogenous tissue at single cell level

Suraj Kannan, Michael Farid, Brian L. Lin, Matthew Miyamoto, Chulan Kwon

Corresponding Author: Chulan Kwon
E-mail: ckwon13@jhmi.edu

This PDF file includes:

Responses to Reviewers
Figs. SR1 to SR6
SI References

Supporting Information Text

We are responding to reviews to our manuscript entitled "Transcriptomic entropy benchmarks stem cell-derived cardiomyocyte maturation against endogenous tissue at single cell level." We thank the reviewers for their thoughtful comments. As our intention is to make both the reviews and our responses publicly available, we wrote these responses to be as comprehensive as possible. We hope that this will both answer some of the concerns raised by the reviewers as well as serve as a resource for the general readership of this manuscript.

Response to Reviewer 1

First, the process of cell dissociation coupled with cell sorting may be associated with a time lag in sample preparation that might be expected to affect RNA stability. If comparisons were performed between scRNA-seq and bulk RNA-seq, would the entropy scores have been equally informative or would differences have been observed from RNA instability that may have affected the entropy scores? While this test would be difficult with *in vivo* acquired cells, such a comparison could have been made using purified (but not sorted) hPSC-CMs. An answer to this question might be valuable to investigators who wish to use your approach to examine existing bulk RNA-seq datasets. Basically, is the workflow only applicable for scRNA-seq data where problems of cell heterogeneity can be eliminated, even though you provide evidence on how to exclude non-CMs from your datasets using transcriptome profiles?

The reviewer raises several important points in this comment that need to be addressed thoroughly. We break down our response to focus on two aspects – 1) the question of cell/tissue dissociation and 2) applicability to bulk RNA-seq data.

Cell/Tissue Dissociation. As the reviewer notes, it is increasingly being appreciated that sample preparation itself can affect measured gene expression. We previously compared live vs fixed CMs after isolation with LP-FACS (1). These samples are included in our entropy meta-analysis (included together as "Kannan Fixed" in **Fig. 4b**), and showed broadly comparable entropy (4.72 ± 0.18 for live, 4.59 ± 0.28 for fixed, comparable to other datasets at the adult timepoint). However, these samples were acquired after Langendorff dissociation; dissociation itself could have led to gene expression changes (2–4). Addressing this issue is challenging - the only approach that we know of to isolate single CMs from previously fixed hearts requires PFA (5), which is incompatible with RNA-seq. We should note that this issue may also affect bulk RNA-seq samples, as many bulk studies may use a dissociation step prior to RNA isolation.

Despite this, we believe that transcriptomic entropy should be generally robust to potential dissociation effects, particularly in CMs. CM maturation affects genes that are generally highly expressed (sarcomeric, mitochondrial, electrophysiological) with large changes over timepoints. By contrast, previously identified dissociation-related stress signatures seem to affect genes that are generally lower expressed and do not change as dramatically over timepoint. Thus, we anticipate that transcriptomic entropy can still stage single CMs with good accuracy despite potential perturbations from dissociation.

Applicability to Bulk RNA-seq. As an example test case, we computed entropy using published isolated cardiomyocyte bulk RNA-seq datasets from Chevalier et al. (6). This study performed sequencing after specifically isolating adult cardiomyocytes. We found the entropy scores for the samples to be 5.06 ± 0.31, comparable to our scRNA-seq results. This suggests that, at least for isolated CMs, bulk and scRNA-seq scores are comparable. However, we acknowledge that more careful benchmarking with more timepoints would be required, though we were unable to find additional qualifying datasets.

The issue of assessing *in vivo* bulk cardiac samples with entropy score is challenging. While CMs (and the CM) signature generally predominates in cardiac tissue RNA-seq, and an entropy decrease can be observed over time, there is inherently some uncertainty that this decrease originates solely from CM maturation. This issue is compounded in PSC-derived cardiac samples, where in addition to heterogeneity of maturation states in CMs, there is also heterogeneity of cell type. Differences in entropy between samples, for example, could be explained by differences in CM purity within the sample. To this end, our recommended usage for transcriptomic entropy as laid out in the manuscript is for scRNA-seq data. For bulk datasets with high (e.g. >95%) purity across all samples, however, we expect transcriptomic entropy to be reasonably comparable. For datasets with lower purity, we may expect that while transcriptomic entropy may not be comparable to our compile single cell reference, it may be useful to compare between samples within the study - so long as the purity is consistent within the study. Thus, for users who cannot perform scRNA-seq, we strongly recommend using an outside measurement of purity and aiming for high between-sample consistency.

As a side note, our approach to eliminating non-CMs was limited to scRNA-seq data. However, recently, several methods have been developed to "deconvolve" bulk RNA-seq data (7). Benchmarking these different methods is somewhat outside the scope of our current work; however, as these tools improve, they may offer an approach for integrating bulk RNA-seq data more effectively with our entropy score.

Second, would mouse strain differences or sex differences cause a shift in the entropy scores or pseudotime analyses, even if only marginally? Not all mouse models develop at the same rate and sex is known to affect

both murine fetal and infant growth.

As noted by the reviewer, all of these factors could lead to changes in the specific timing of the entropic trajectory over CM maturation. Even within a litter, individual mice develop at different rates. As an example, we plot entropy scores for three biological replicates animals at several timepoints within one dataset **(Fig. SR1)**. Given the fact that different studies often used different strains, this may explain minor differences in entropy score between studies, though it would be impossible to deconvolve this variable from other differences between studies.

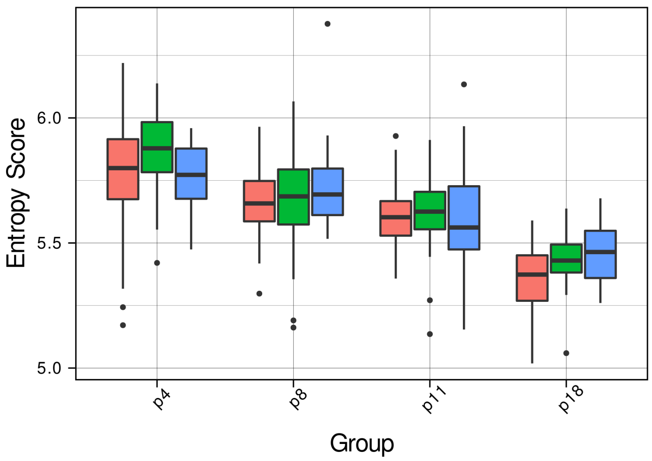

Fig. SR1. Entropy score for multiple animals at select timepoints within one dataset. We included at least one male and one female at each timepoint, though unfortunately do not have labeled which animal was which, so we cannot conclude specific sex-based differences at this time.

However, as suggested by the reviewer, we anticipate these differences tend to be marginal compared to the large-scale changes associated with maturation. To use our example here, the spread of cell maturation within an animal and the differences between timepoints are both larger than the differences between individual animals. Likewise, we don't expect the genes that become differentially expressed to change widely, though the specific timing of their changes may be somewhat accelerated or delayed.

Third, when performing the entropy scores and pseudotime analyses, were there specific transcripts or groups of transcripts that were more informative of specific stages of maturation? You mention that ∼81.5% were identified as differentially expressed by all methods and some transcript profiles are shown in Figure 4e, but were any informative genes or gene sets (i.e., markers) more useful for assessing maturation that would not require scRNA-seq? This information (which could be added in the supplement) might make your approach more accessible to the broader research community (i.e., the identification of new and informative markers of CM development or differentiation). Alternatively, it may be that scRNA-seq is required. If so, then this should be discussed.

The focus of our manuscript was on developing a method to stage CMs across scRNA-seq studies, particularly for benchmarking PSC-CMs. However, as noted by the reviewer, developing an improved understanding of *in vivo* maturation is important, both on its own merits and to identify better markers for studying PSC-CM maturation. Here, we would actually direct reviewers to a more recent study of ours, which touches upon these issues (8). In that study, we extended the *in vivo* reference generated in this manuscript, and thoroughly classified differentially expressed genes by their temporal expression patterns. In particular, we identified how genes change across the embryonic/neonatal and perinatal time periods identified in this manuscript. We should note that while several of the results in this newer manuscript were generated using traditional dimensionality reduction approaches, similar results could be obtained using entropy score given the strong concordance between the methods.

The applications of marker-based analysis should also be considered. One effective use of a panel of marker genes would be to see, for example, if PSC-CMs improve in maturation status in a biomimetic manner. However, the advantage of entropy score is its ability to compare cross-study, both in terms of benchmarking PSC-CMs to endogenous cells and across multiple PSC-CM datasets. In our experience, we have found qPCR to produce variable results across machines and labs, analogous to RNA-seq batch effects. Thus, even with a robust panel, it would likely be difficult to similarly benchmark qPCR results across different research groups. Of course, it is possible that we may be able to define a small panel of markers, perform qPCR, and then compute entropy on those measurements. However, testing this rigorously is beyond the scope of this manuscript.

Finally, could you comment further on the application of entropy scores to study maturation and how your approach may be of value to the research community? A number of situations beyond comparisons

of engineered and *in vivo* tissues, and somatic cell reprogramming protocols might include an evaluation of PSC-CMs for pharmaceutical and toxicity testing, and the prediction of pathways that may be essential for maturation of cells either through a gene regulatory network or through individual signaling pathways. While these experiments and discussion points are not necessary to support your conclusions, an evaluation of these points and limitations in the Discussion may broaden the paper's impact and significance.

We appreciate this point from the reviewer. One particular area where we believe entropy score may be valuable is to facilitate understanding of regulators of *in vivo* CM maturation. There has been recent interest in studying cell-autonomous regulators of CM maturation through mosaic knockout systems (9, 10). Our group recently used this approach to study the roles of PGC1 in endogenous CM maturation (11). As an example, we show below the computed entropy scores for PGC1α/β KO cells compared with control cells **(Fig. SR2)**. As can be seen, a maturation defect initiates around $\sim$ P14 and is particularly prominent by P28. More recently, others have applied forward genetic screens using mosaic systems to identify regulators of CM maturation (12). However, identifying an optimal readout for such screens is challenging. We anticipate that entropy score may be useful in these contexts, particularly where extensive timepoint data may not be available. Using optimized protocols for high-throughput knockout screening followed by scRNA-seq, researchers could use entropy score to identify previously unstudied regulators of CM maturation. Of course, this same approach could be used with PSC-CMs to, for example, screen potential activators of *in vitro* maturation. We have included further discussion of these possibilities into our Discussion section.

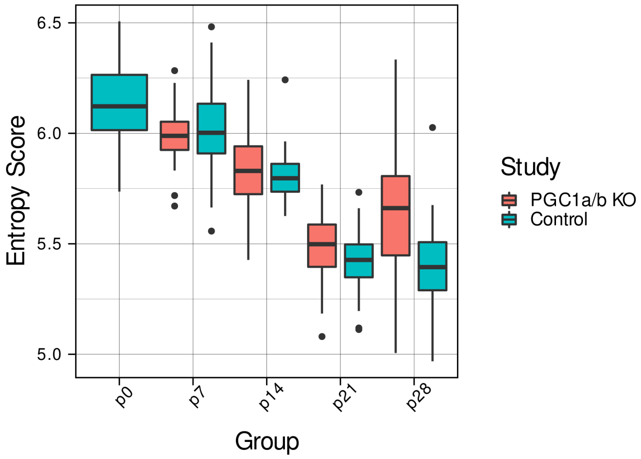

Fig. SR2. Entropy score across control and PGC1 α/β KO from Murphy et al. Cells were generated by using viral delivery of Cre to ensure a mosaic system in which cell-autonomous impacts of knockout could be studied.

With regards to studying disease processes or toxicity, we direct readers to our comments below to Reviewer 2 about non-maturation-related applications of entropy score. However, we urge some caution with interpretation. We focused our analyses on the maturation process specifically, and would need to perform more rigorous testing to study changes to entropy score in specific disease processes.

Response to Reviewer 2

It may be useful to emphasize that matching the entropy score of *in vivo* cardiomyocytes (or a given CM developmental state) is not a sufficient indication of matching the expression patterns of the *in vivo* counterpart.

This is a really important point identified by the reviewer; we have included some further discussion of this in the second paragraph of the discussion. Our recent manuscript (8) touches on this issue further, and we direct interested readers to our results there. As a quick summary, however, PSC-CMs may indeed have a comparable transcriptomic entropy or be matched to a given CM developmental state by dimensionality reduction methods while still showing important expression differences. Nevertheless, entropy score provides an approximation of the overall maturation stage of the cell.

Compare entropy scores from cardiomyocytes from snRNA-seq on post mortem tissue (Litviňuková, et al).

The Litviňuková et al. dataset (13) provides an interesting set of considerations. As discussed in the manuscript, we found that most nuclear datasets presented with overly low depth, well below our thresholds established in **Fig. 3b**. Moreover, sensitivity (e.g. number of genes detected per cell) was often low, below our standard of 1000 genes/cell. The Litviňuková et al. dataset is notably higher quality, with a median of 1545 genes/cell, though a depth that is still close to our low-end cutoff (3621

counts/cell). However, there is another issue that must be considered with nuclear datasets, in that they may fundamentally present with different gene distributions than whole cell datasets simply because of differences in RNA capture. There are several ways to note this. For one, we have observed that the ratio of counts:genes is higher in nuclear datasets compared to whole cell datasets (not shown here), inherently suggesting a different distribution of counts (with many more "single count" genes in nuclear datasets). Moreover, entire classes of genes show differences. As a clear example, mitochondrially-encoded transcripts will not be identified in nuclear datasets, while nuclear lncRNAs (most notably *Malat1*) will be enriched in nuclear datasets. This issue is further discussed in Selewa et al. (14). The fundamental assumption underlying using transcriptomic entropy to measure maturation status cross-dataset is that gene distributions should remain consistent across different samples at similar maturation states. However, this assumption is violated by nuclear-seq protocols. We anticipate that transcriptomic entropy could be more effectively extended to nuclear datasets once a comprehensive nuclear reference of mouse and human CM maturation (of comparable quality to the Litviňuková dataset) can be assembled, as we have done here with whole cell data. In the interim, we do not encourage direct comparison of nuclear datasets to whole cell datasets with transcriptomic entropy.

Nevertheless, some interesting analysis can be made *within* the dataset. We explored transcriptomic entropy across a sample of the ventricular CMs generated in the study **(Fig. SR3)**. Interestingly, we did find a decrease in entropy score over timepoint (**Fig. SR3a**), despite the fact that all the CMs came from ostensibly mature hearts. However, this may be confounded with the source of the nuclei - in general, older samples came from the Sanger Institute (**Fig. SR3b**), and these samples systematically presented with higher percentages of *MALAT1* (**Fig. SR3c**, potentially indicative of large sample isolation differences). This may also further indicate the need to develop unique gene filtration strategies for nuclear RNA-seq data, as we did in the manuscript for whole cell data. Notably, there were no differences in transcriptomic entropy across the various CM subpopulations identified by the authors **Fig. SR2d**.

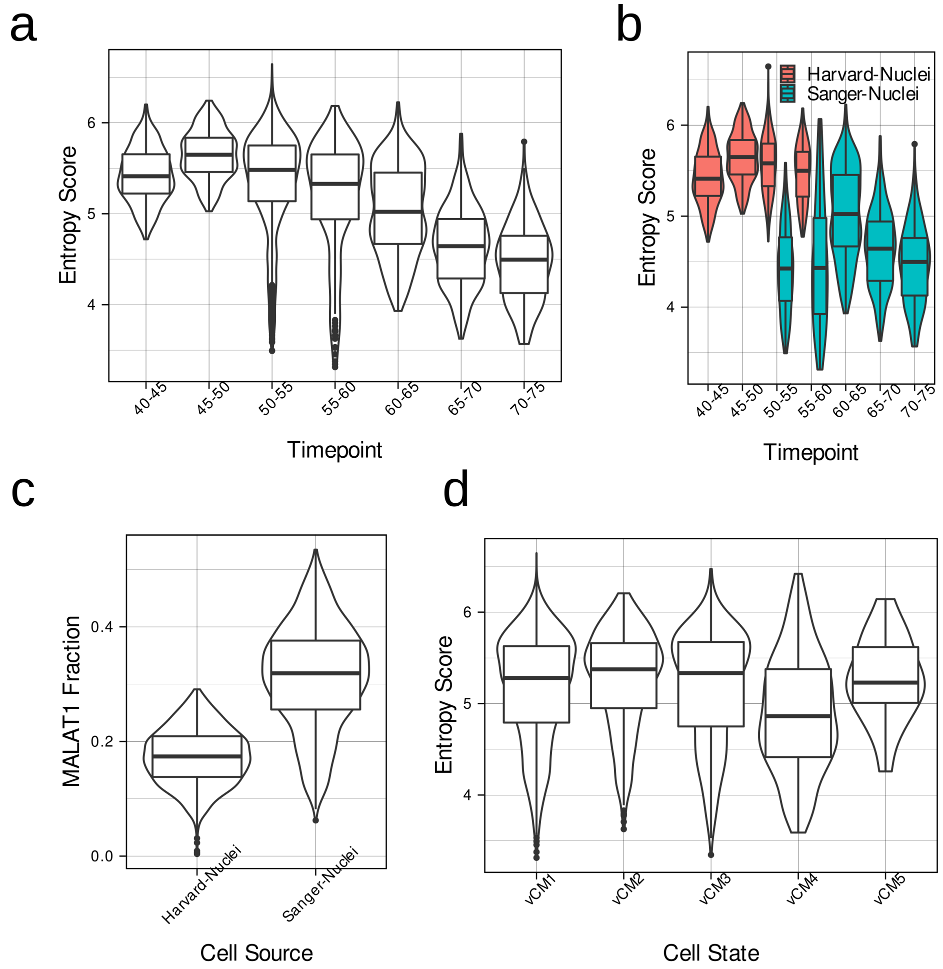

Fig. SR3. Analysis of ventricular CM nuclear RNA-seq data from Litviňuková et al. a. Entropy scores by age. **b.** Entropy scores by age, further labeled by source of nuclei. **c.** Fraction of reads assigned to *MALAT1*, a nuclear lncRNA, based on source of nuclei. **d.** Entropy score across the ventricular CM states identified by the authors.

There are differences in cardiomyocytes obtained from different regions of the human heart (atrial vs. ventricular, left vs. right, etc.). It will be informative to compare the many *in vitro* differentiation datasets (and protocols)

that may give result in atrial-like or ventricular-like CM to their *in vivo* counterparts.

The reviewer raises a particularly interesting point here - while it is well known that CMs show regional variations (even within the same cardiac chamber), the degree to which the maturation process varies across the chambers is unknown. Within our study, our generated perinatal maturation reference was isolated solely from LV CMs. However, when performing our meta-analysis of *in vivo* datasets, we included CMs from other anatomical locations as well, including RV and both atria. Specific details about the datasets can be found in the **Appendix**. Notably, despite comparing a fairly heterogeneous mix of CMs, we observed a generally consisting trend in entropy. This may suggest that despite specific molecular differences across chambers, maturation itself may be relatively well-conserved. Thus, we are optimistic that entropy score can be used to consistently study maturation in CMs regardless of anatomical origin. However, we acknowledge that this must be characterized more systematically in the future.

With regards to PSC-CMs, the question becomes more complicated. Protocols have been developed to push cells towards a more atrial-like or ventricular-like phenotype, though to date we have not observed any scRNA-seq datasets generated from these protocols. We focused our attention on PSC-CMs generated using standard protocols, which may generate a mix of phenotypes. Characterizing the chamber status of these PSC-CMs is complicated. We and others have reported that molecular markers of chamber status are often inconsistent in PSC-CMs (8, 15), in particular because canonical atrial markers are also expressed in immature ventricular CMs *in vivo*. The gold standard for subtyping PSC-CM phenotype is patch clamping, but a) this does not always yield clear distinctions in phenotype and b) is currently incompatible with scRNA-seq. We suspect that further methods to assess CM subtype will be necessary to more comprehensively classify PSC-CMs.

This question pertains to *in vitro* CM differentiation: Is entropy score sensitive to cell-types that differentiate into alternative lineages during *in vitro* differentiation (issue of purity)? Different cell lineages may have different maturation rates and if they are not excluded, the non-cardiomyocyte cells could contribute to noisy measurements. If the entropy score is calculated after a first round of clustering, on identified CM among the population (as opposed to cardiac progenitor cells, for example), I would be more confident of the entropy score.

As noted by the reviewer, we anticipate that different cell types may have different maturation rates and thus different entropy scores. Thus, for all of our analyses involving PSC-CMs, we used the sample filtration approach as we did with our *in vivo* CM samples. Specifically, this including a cell type classification step using SingleCellNet. The results for PSC-CMs shown in the manuscript only include cells classified as CMs. (Please also note that specific studies may have had other filtration steps – more details for each study can be found in the **Appendix**). Likewise, because our analysis of PSC-CMs begins at D9 of differentiation, we anticipate that we have CMs rather than multipotent progenitors.

This also pertains to *in vitro* CM differentiation: Even within the cardiomyocyte lineage, there may be different rates of development that ultimately lead to the same end point. Therefore there may be the need to coarse-grain the developmental time-points to account for the precocious ones and the 'late bloomers'. It may be useful to anchor the developmental trajectory based on entropy score to biological milestones (such as when the CMs start beating in plates). Can the authors comment on this, please?

We agree with the reviewer that this question of differential rate of maturation is an interesting one. Both this manuscript as well as several others (8, 11, 16) have identified significant heterogeneity of maturation rates at the single cell level, despite eventual convergence to an adult state. The idea of anchoring to some other cell phenotypic readout is intriguing, though the challenge is in identifying the appropriate readout. For example, our focus is on CM maturation after specification. However, CMs begin to beat more or less immediately after specification - *in vitro*, PSC-CMs may begin to beat between D6-D7 of differentiation (depending on the protocol/cell line), while our analysis here already begins after that timepoint. We have also briefly looked at PSC-CM size. In our recent work (8), we observed a population of larger PSC-CMs emerging at D25 of *in vitro* differentiation. Surprisingly, however, we noted few transcriptional differences between larger and smaller cells, though similar results have also been seen *in vivo* (17). Another interesting recently identified candidate is CD36 (18). We anticipate that entropy score can be a useful tool for helping identify candidate milestones/phenotypic characteristics to stratify CMs by developmental rate.

CMs are interesting in that they are post-mitotic and as such, will attain a level or maturity at the end of the maturation process. I can imagine this not being the case for cells that continue to cycle and divide. It would be interesting to compare the change in entropy score for such cells. How about cells that differentiate when activated by an external stimulus (e.g., immune cells)? As long as a cell has high transcriptional variability or is transcriptionally active (e.g., as stress response) it may still show high entropy score. How would one interpret Entropy scores in such situations?

The focus of our analysis here was to generate a method to compare maturation states across single cell RNA-seq datasets, where maturation was implicitly defined based on the assumption of eventual quiescence (or approximate quiescence, in the case of

 Suraj Kannan, Michael Farid, Brian L. Lin, Matthew Miyamoto, Chulan Kwon

liver/pancreas). The rationale for this focus was the practical observation that PSC-derived tissues aiming to recapitulate these largely quiescent organs have remained immature. However, as noted by the reviewer, there are many intriguing applications of transcriptomic entropy outside the scope of our analysis. Indeed, transcriptomic entropy was initially used to assess stem cell potency during differentiation processes. During our pilot studies (not shown here), we observed a dataset of early stage neuronal development in which entropy zigzagged as cells went through progressive stages of specification. We also guide readers to other studies of interest that have used similar entropic measures to study different aspects of biology. For example, Grun et al. studied entropy changes over the course of hematopoiesis (19). The same study also investigated a population of putatively dedifferentiated ductal progenitor cells in adult pancreas. The Teschendorff group has also used a signaling entropy metric to identify cancer cells among normal tissue - as the normal tissue becomes perturbed, the entropy correspondingly increases (20, 21).

Given these various applications, we would suggest that entropy score be carefully interpreted to the context of the data being compared. However, as CM maturation is a notably unidirectional process with a clearly defined endpoint, entropy score should robustly allow for quantification of this process.

The authors note "higher mtGENE in differentiated cells and later time points." - Fig 2a. Could this be related to difficulty in dissociation, as part of stress response?

We have found that interpreting the mitochondrial read percentage in CM datasets is somewhat tricky. On the one hand, we note that whole heart bulk RNA-seq datasets (with no dissociation step) show high percentages of mitochondrial RNA reads compared to other tissues. This issue, for example, has been discussed by Mercer et al. (22), and more recently by Zhou (23). This also matches with expectations, as CMs are highly mitochondria rich, and increase in number and size of mitochondria is associated with CM maturation. Thus, we anticipate that the increasing mitochondrial percentage over timepoint (up to ∼45% in adult CMs) is likely biological. On the other hand, excessively high mitochondrial read percentages (as indicated in **Fig. 3a**) are likely due to cell damage (leading to loss of cytoplasmic but not mitochondrial RNAs) or stress response. (More details can be found at (24).)

The authors note "In particular, 10x Chromium and STRT-seq datasets appeared to have systematically higher percentages of ribosomal protein-coding genes than other protocols." Could this simply be due to higher transcript capture rate of these protocols? These protocols/techniques may not be statistically sampling a cell's transcripts at the same rate as the techniques with "lower" capture efficiency.

While this is possible, it should be noted that the Smart-seq2 and mcSCRB-seq protocols are currently the most sensitive library preparation protocols. In particular, mcSCRB-seq has been optimized for high transcript capture compared to other protocols (25). However, both of these protocols show generally low ribosomal protein-coding gene rates. Anecdotally, we have heard from others about high ribosomal protein-coding genes in 10x Chromium data, and the percentages suggested by the company themselves are notably high (https://kb.10xgenomics.com/hc/en-us/articles/218169723-What-fraction-of-reads-map-to-ribosomal-proteins-). It is possible that this phenomenon has more to do with the binding properties of the specific primers used in the 10x Chromium protocol, leading to a systematic bias, but this would need to be further studied. As many studies treat ribosomal protein-coding genes as contaminants or uninformative, we proceeded similarly and filtered them from consideration.

Can entropy score be used in the context of activation (under external stimulus) or deactivation (when the external stimulus is removed)?

We hope that entropy score will be useful for this exact case, especially in the context of aiming to generate more mature PSC-CMs through application of external stimulus. To date, there have been few scRNA-seq datasets of PSC-CM perturbations, and our own dataset collection was unfortunately interrupted by the pandemic. However, as a test case, we applied entropy score to two recent perturbation datasets of PSC-CMs **(Fig. SR4)**. The first approach, generated by Giacomelli, Meraviglia, and Campostrini et al., used co-culture of PSC-CMs with non-CMs (26). This approach yielded limited change in entropy score (**Fig. SR4a**). The second study, from Lam et al., used both anistropic patterning and tissue engineering (27). While this approach yielded a decrease in entropy score over control with both methods, the end CMs still failed to achieve a maturity greater than corresponding fetal human tissue or D100 PSC-CMs (**Fig. SR4b**).

What do the black dots represent in Fig 2c?

These were simply outliers on the box-and-whiskers plot. We apologize for the confusion - because of a graphic error the outlier points were plotted to be extremely large. We have corrected this in the updated draft.

Response to Reviewer 3

The calculation of the entropy is not clear enough (or not performed correctly). Shouldn't P_i be the GeX distribution of Gene i across all cells? The authors seem to have calculated P_i as the probability of expression in one cell then summed across. Unless I am wrong, this does not make sense and invalidates all the analysis.

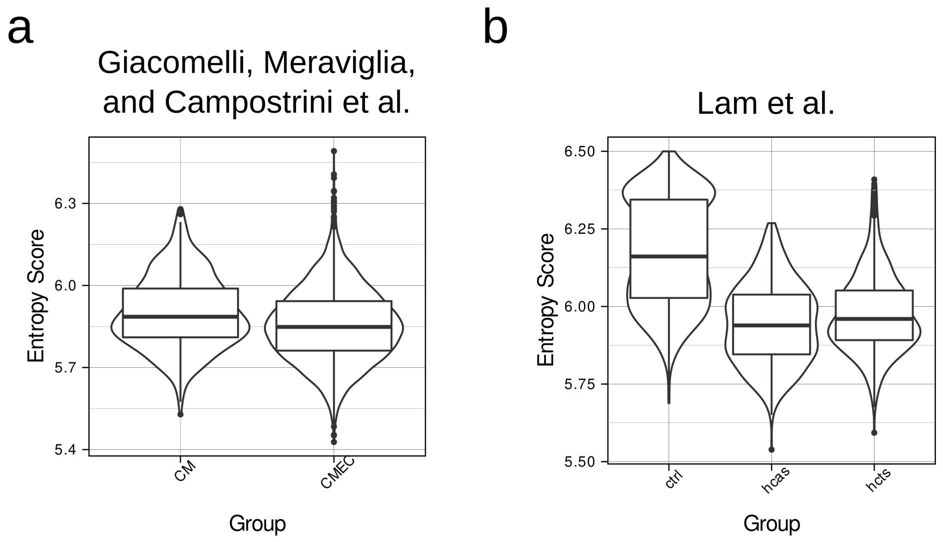

Fig. SR4. Analysis of tissue engineered approaches for improving PSC-CM maturation. a. Entropy scores for samples in Giacomelli, Meraviglia, and Campostrini et al. The test sample (CMEC) contains co-cultured PSC-CMs and PSC-derived endothelial cells. **b.** Entropy scores for samples in Lam et al. The control samples were cultured in normal monolayer, while the test samples came from one of two engineering approaches - an anisotropic sheet (hcas) or an engineered heart microtissue using microposts (hcts).

Also P_i being a probability, how was the normalization performed so that the sum of the probability is 1? Given the variability in gene expression, scRNAseq platforms and number of cells it would be good to have a metric estimating the quality of the distribution.

We appreciate the chance to further clarify the rationale behind our approach. Our definition of transcriptomic entropy draws from other studies. The premise is to use Shannon entropy as a diversity index of gene expression in a single cell. An immature cell will have generally higher diversity - more genes expressed, and cell-type specific genes expressed at lower levels. Conversely, a mature cell will have lower diversity - fewer genes with higher expression in genes critical for cell function. This diversity is computed on the single cell level - e.g., what is the distribution of genes within one single cell? Thus, we compute entropy for a single cell by using the probability of each gene within the cell (which is effectively just a scaled expression value of the gene). P_i is thus the probability of selecting a count of a particular gene from the bag of all gene counts within a cell. We can then compute $P_i \log(P_i)$ for each gene and sum for the single cell (though, as discussed in the manuscript, our eventual approach subset to only the top 1000 expressed genes in each cell). What we are interested in is the distribution for the single cell, rather than the distribution of a particular gene across a population of cells. This approach led to several advantages. Firstly, entropy score can be calculated using information within just one cell. Thus, unlike dimensionality reduction methods, which rely on relationships between cells, entropy can be computed effectively on datasets with fewer cells or perhaps only one biological timepoint (and therefore possibly less variation in specific genes across that dataset). The second benefit is that entropy score does not rely on any on particular gene or set of genes. This fact was critical in enabling cross-study comparisons. We and others have generally observed that expression of specific genes is not stable across multiple studies. Thus, a method that relies on directly comparing expression levels will inevitably be complicated by batch effects - indeed, this is likely why trajectory reconstruction methods do not fully enable cross-study comparisons. By contrast, by relying on the overall gene distribution rather than a predefined set of genes, entropy score facilitates cross-study comparison.

We hope this also answers another question regarding normalization. Entropy simply normalizes genes within a cell by dividing by the total counts within the cell (effectively analogous to CPM normalization). This gives a probability that will sum to 1.

Entropy score correlated only moderately with pseudotimes for the three methods. This is a major problem that needs to be explained. One would expect entropy to give a higher correlation if it is a robust measure of development.

We thank the reviewer from bringing up this point - however, there are several important caveats to note. Firstly, we believe it is erroneous to assume that trajectory reconstruction methods are inherently a gold standard for single cell development. Trajectory reconstruction methods carry their own assumptions, namely the validity of dimensionality reduction as an approach for capturing gene expression changes over dynamic processes. This assumption does not necessarily always hold (28). Individual trajectory reconstruction methods may also carry their own specific variations and assumptions, which

will also change their downstream predicted pseudotimes. Given that transcriptomic entropy is based on a completely different framework, without reference to dimensionality reduction or even intra-cell distances, we are not surprised to see some variation between the methods. This may compounded by single cell noise, where cells with very close transcriptional states may be placed in slightly different orders relative to one another across different methods.

We further note that entropy shows generally strong concordance with pseudotime methods during the range of embryonic and perinatal CM maturation before deviating more significantly from pseudotime methods once maturity is reached. We further validated this observation using a more extensive CM maturation reference from our recent study (8) (**Fig. SR5**). We again expect that these variations in the ordering of late-stage CMs are due to inherently different assumptions in the methods. However, given the good concordance during the critical perinatal period of CM maturation, during which the dynamic range is most biologically important, we believe that entropy is effective for quantifying maturation status. Moreover, the high concordance in identified differentially expressed genes between entropy and pseudotime methods further supports the fact that they are capturing a similar biological maturation process.

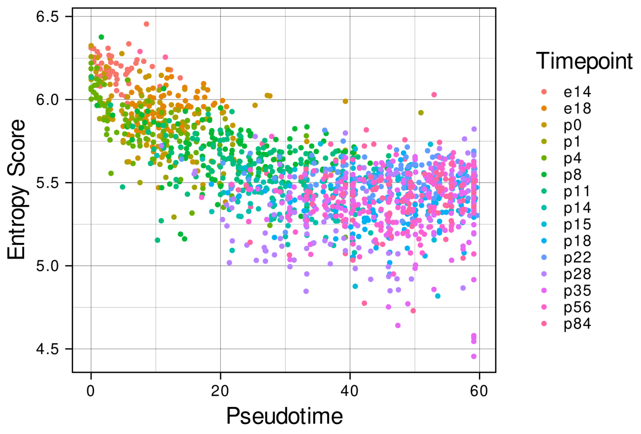

Fig. SR5. Entropy score vs pseudotime for CM maturation reference in Kannan et al., 2021. Pseudotime was computed using Monocle 3.

One of the main purposes of the approach is to classify maturation of *in vitro* datasets, but basically no entropy changes are found. They are minimal in Figure 5c. Following with this, the developmental times of the datasets as shown by color codes do not match the changes in entropy (see Figs 4b, 5a/b).

As noted by the reviewer, one of our main motivations for developing entropy score was to classify PSC-CM maturation. **Fig. 5a** is somewhat tricky to interpret, as unlike *in vivo*, where there is a consistent maturation program, PSC-CM maturation is also confounded by differences in protocols and cell lines. Thus, while there are broad changes in maturation between, for example, D9-12 vs D45-D100, cells from different datasets will proceed at slightly different rates. More clear differences across timepoint can be seen by looking at each study individually. We also agree with the observation by the reviewer that the range of maturation in PSC-CMs **(Fig. 5a)** and reprogrammed CMs **(Fig. 5b)** is minimal. This is, we believe, a key finding from the study. It is an assumption that PSC-CMs are undergoing a maturation process *in vitro* - however, our belief is that any maturation process that is happening is exceedingly minimal, particularly in comparison to the scope of changes that are happening *in vivo*. Even late-stage PSC-CMs fail to achieve greater than a fetal-like entropy score, and appear arrested at the onset of the *in vivo* perinatal phase. This observation is consistent with data from numerous other studies, and indeed underlies the observation that PSC-CMs are immature. In our recent study (8), we probed this observation more thoroughly, and found that mouse and human PSC-CMs generally fail to undergo the perinatal CM maturation programs that happen *in vivo*. We believe the data in this manuscript are consistent with these findings.

Regarding the color codes in the two mentioned figures - the colors simply reflect the source of the data (e.g. which study). The purpose of color-coding in this manner was to demonstrate consistency of entropy scores across studies, particularly *in vivo*.

Why is the entropy not compared between the Kannan dataset and Wang and Yao? This would prove that indeed entropy is a good measure as opposed to UMAP+monocle.

We apologize for the confusion here - both of these datasets are included in **Fig. 4b**. We show a comparison below here as well **(Fig. SR6)**. Given that we have observed maturation states to overlap significantly from e18 - p4 (8, 16), we believe these results match our expectations.

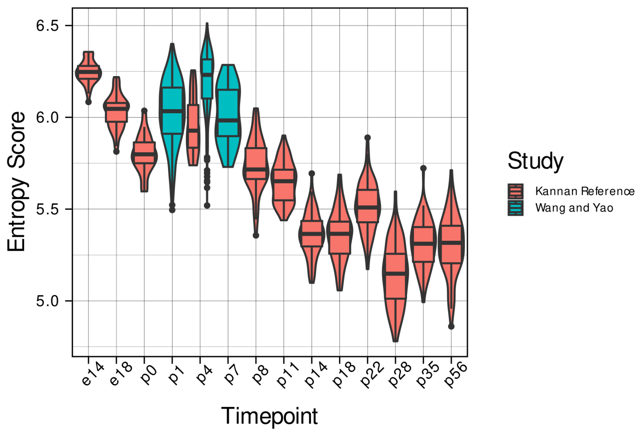

Fig. SR6. Entropy score across timepoints for Wang and Yao et al. and the maturation reference generated in this manuscript.

References

1. S Kannan, C Kwon, Regulation of cardiomyocyte maturation during critical perinatal window. *J. Physiol.* **0**, 1–16 (2019).
2. SC Van Den Brink, et al., Single-cell sequencing reveals dissociation-induced gene expression in tissue subpopulations. *Nat. Methods* **14**, 935–936 (2017).
3. E Denisenko, et al., Systematic assessment of tissue dissociation and storage biases in single-cell and single-nucleus RNA-seq workflows. *Genome Biol.* **21**, 1–25 (2020).
4. L Machado, et al., Tissue damage induces a conserved stress response that initiates quiescent muscle stem cell activation. *Cell stem cell*, 1–11 (2021).
5. D Yücel, J Solinsky, JH van Berlo, Isolation of cardiomyocytes from fixed hearts for immunocytochemistry and ploidy analysis. *J. Vis. Exp.* **2020**, 1–17 (2020).
6. M Chevalier, et al., Transcriptomic analyses of murine ventricular cardiomyocytes. *Sci. Data*, 1–9 (2018).
7. F Avila Cobos, J Alquicira-Hernandez, JE Powell, P Mestdagh, K De Preter, Benchmarking of cell type deconvolution pipelines for transcriptomics data. *Nat. Commun.* **11** (2020).
8. S Kannan, M Miyamoto, BL Lin, C Kwon, Trajectory reconstruction identifies dysregulation of perinatal maturation programs in pluripotent stem cell-derived cardiomyocytes. *bioRxiv* (2021).
9. Y Guo, et al., Analysis of Cardiac Myocyte Maturation Using CASAAV, A Platform for Rapid Dissection of Cardiac Myocyte Gene Function In Vivo. *Circ Res.* **120**, 1874–1888 (2018).
10. Y Guo, et al., Hierarchical and stage-specific regulation of cardiomyocyte maturation by serum response factor. *Nat. Commun.* (2018).
11. S Murphy, et al., PGC1 / PPAR Drive Cardiomyocyte Maturation through Regulation of Yap1 and SF3B2. *bioRxiv* (2020).
12. NJ VanDusen, et al., In vivo CRISPR screening identifies RNF20/40 as epigenetic regulators of cardiomyocyte maturation. *bioRxiv* (2019).
13. M Litvinuková, et al., Cells of the adult human heart. *Nature* **588**, 466–472 (2020).
14. A Selewa, et al., Systematic Comparison of High- throughput Single-Cell and Single- Nucleus Transcriptomes during Cardiomyocyte Differentiation. *Sci. Reports* **10**, 1–13 (2020).
15. NT Feric, M Radisic, Maturing human pluripotent stem cell-derived cardiomyocytes in human engineered cardiac tissues . *Adv. Drug Deliv. Rev.* **96**, 110–134 (2016).
16. DM DeLaughter, et al., Single-Cell Resolution of Temporal Gene Expression during Heart Development. *Dev. Cell* **39**, 480–490 (2016).
17. M Yekelchyk, S Guenther, J Preussner, T Braun, Mono - and multi - nucleated ventricular cardiomyocytes constitute a transcriptionally homogenous cell population. *Basic Res. Cardiol.* **114**, 1–13 (2019).
18. ENY Poon, et al., The cell surface marker CD36 selectively identifies matured, mitochondria-rich hPSC-cardiomyocytes. *Cell Res.* **30**, 626–629 (2020).
19. D Grun, et al., De Novo Prediction of Stem Cell Identity using Resource De Novo Prediction of Stem Cell Identity using Single-Cell Transcriptome Data. *Cell Stem Cell* **19**, 266–277 (2016).
20. AE Teschendorff, T Enver, Single-cell entropy for accurate estimation of differentiation potency from a cell's transcriptome. *Nat. Commun.* **8**, 1–15 (2017).
21. W Chen, AE Teschendorff, Estimating Differentiation Potency of Single Cells Using Single-Cell Entropy (SCENT). *Comput. Methods for Single-Cell Data Analysis* **1935**, 125–139 (2019).
22. TR Mercer, et al., The Human Mitochondrial Transcriptome. *Cell* **146**, 645–658 (2011).
23. B Zhou, L Wang, Reading the heart at single-cell resolution. *J. Mol. Cell. Cardiol.* **148**, 34–45 (2020).

24. S Kannan, et al., Large particle fluorescence-activated cell sorting enables high-quality single-cell RNA sequencing and functional analysis of adult cardiomyocytes. *Circ. Res.* (2019).
25. JW Bagnoli, et al., Sensitive and powerful single-cell RNA sequencing using mcSCRB-seq. *Nat. Commun.* **9** (2018).
26. E Giacomelli, et al., Human-iPSC-Derived Cardiac Stromal Cells Enhance Maturation in 3D Cardiac Microtissues and Reveal Non-cardiomyocyte Contributions to Heart Disease. *Cell Stem Cell* **26**, 862–879.e11 (2020).
27. YY Lam, et al., Single-Cell Transcriptomics of Engineered Cardiac Tissues From Patient-Specific Induced Pluripotent Stem Cell-Derived Cardiomyocytes Reveals Abnormal Developmental Trajectory and Intrinsic Contractile Defects in Hypoplastic Right Heart Syndrome. *J. Am. Hear. Assoc.* **9**, e016528 (2020).
28. SM Cooley, T Hamilton, EJ Deeds, JCJ Ray, A novel metric reveals previously unrecognized distortion in dimensionality reduction of scRNA-Seq data. *bioRxiv*, 1–36 (2019).
